# Discovery of Orexant and Anorexant Agents with Indazole Scaffold Endowed with Peripheral Antiedema Activity

**DOI:** 10.3390/biom9090492

**Published:** 2019-09-16

**Authors:** Marilisa P. Dimmito, Azzurra Stefanucci, Stefano Pieretti, Paola Minosi, Szabolcs Dvorácskó, Csaba Tömböly, Gokhan Zengin, Adriano Mollica

**Affiliations:** 1 Department of Pharmacy, University of Chieti-Pescara “G. d’Annunzio”, Via dei Vestini 31, 66100 Chieti, Italy; marilisa.dimmito@unich.it (M.P.D.); a.mollica@unich.it (A.M.); 2 Istituto Superiore di Sanità, Centro Nazionale Ricerca e Valutazione Preclinica e Clinica dei Farmaci, Viale Regina Elena 299, 00161 Rome, Italy; stefano.pieretti@iss.it (S.P.); Paola.minosi@iss.it (P.M.); 3 Institute of Biochemistry, Biological Research Centre of the Hungarian Academy of Sciences, Temesvári krt. 62, 6726 Szeged, Hungary; dszabolcsbiol@gmail.com (S.D.); tomboly.csaba@brc.mta.hu (C.T.); 4 Department of Biology, Science Faculty, Selcuk University, 42005 Konya, Turkey; gokhanzengin@selcuk.edu.tr

**Keywords:** cannabinoid receptor, rimonabant, food intake, anorexant agent, edema

## Abstract

The endocannabinoid system represents an integrated neuronal network involved in the control of several organisms’ functions, such as feeding behavior. A series of hybrids of 5-(4-chlorophenyl)-1-(2,4-dichloro-phenyl)-4-methyl-*N*-(piperidin-1-yl)-1*H*-pyrazole-3-carboxamide (mimonabant), a well-known inverse agonist of the type-1 cannabinoid receptor (CB1), once used as an antiobesity drug, and the *N*-(2*S*)-substitutes of 1-[(4-fluorophenyl)methyl]indazole-3-carboxamide with 1-amino-3-methyl-1-oxobutane (AB-Fubinaca), 1-amino-3,3-dimethyl-1-oxobutane (ADB-Fubinaca), and 3-methylbutanoate (AMB-Fubinaca), endowed with potent agonistic activity towards cannabinoid receptors CB1 and CB2 were in solution as *C*-terminal amides, acids, methyl esters and *N*-methyl amides. These compounds have been studied by binding assays to cannabinoid receptors and by functional receptor assays, using rat brain membranes in vitro. The most active among them as an agonist, (S)-1-(2,4-dichlorobenzyl)-*N*-(3,3-dimethyl-1-(methylamino)-1-oxobutan-2-yl)-1H-indazole-3-carboxamide (LONI11), and an antagonist, (S)-2-(1-(2,4-dichlorobenzyl)-1H-indazole-3-carboxamido)-3-methylbutanoic acid (LONI4), were tested in vivo in mic, to evaluate their ability to stimulate or suppress feeding behavior after intraperitoneal (i.p.) administration. For a LONI11 formalin test and a tail flick test after an administration by the subcutaneous (s.c.) and intracerebroventricular (i.c.v.) routes, respectively, were also carried out in vivo in mice to investigate the antinociceptive property at the central and peripheral levesl. We observed a significant orexant effect for LONI11 and an intense anorexant effect for (S)-methyl 2-(1-(2,4-dichlorobenzyl)-1H-indazole-3-carboxamido)-3,3-dimethylbutanoate (LONI2) and LONI4. In zymosan-induced edema and hyperalgesia, LONI11 reduced the percent of paw volume increase and paw latency after s.c. administration, also suggesting a possible peripheral anti-inflammatory activity.

## 1. Introduction

**Scheme 1:**
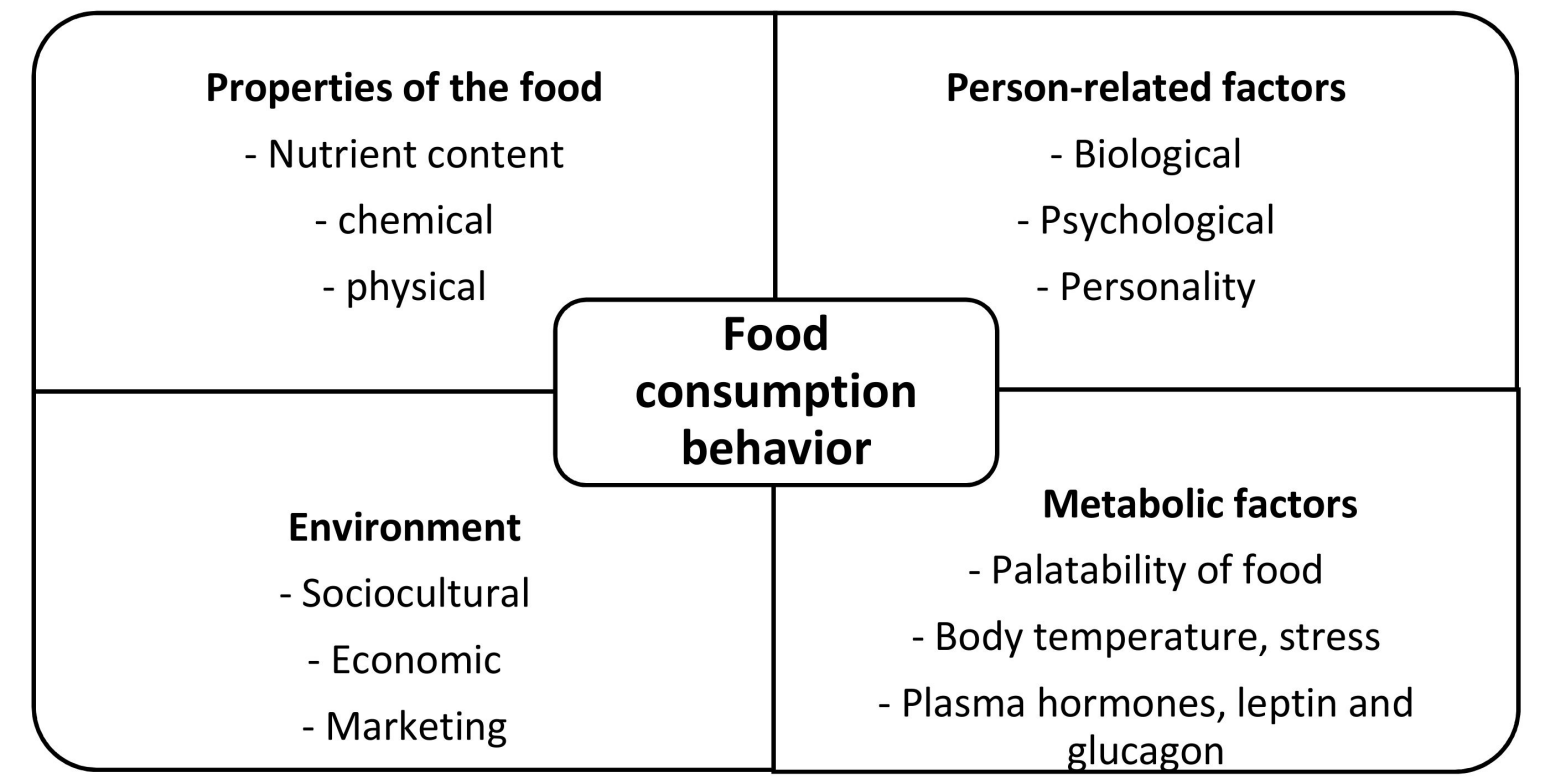
Metabolic and cultural factors involved in the regulation of food intake.

Metabolic syndrome is the result of a group of multifactorial conditions, characterized by the loss of balance between energy income and caloric needs. An efficacious equilibrium between orexigenic and anorexigenic signals ensures a mild feeding behavior, whereas a delicate and growing interference in neurochemistry is enough to provoke its alteration. Motivational feeding behavior is the base of human beings’ underlying food intake to obtain energy from food. This is strongly associated with the concept of “food grabbing,” which is influenced by parents’ food-choice strategies, behavioral contexts, and dietary quality. Metabolic and cultural factors involved in the regulation of food consumption and food intake are reported below (Scheme 1).

**Figure 1:**
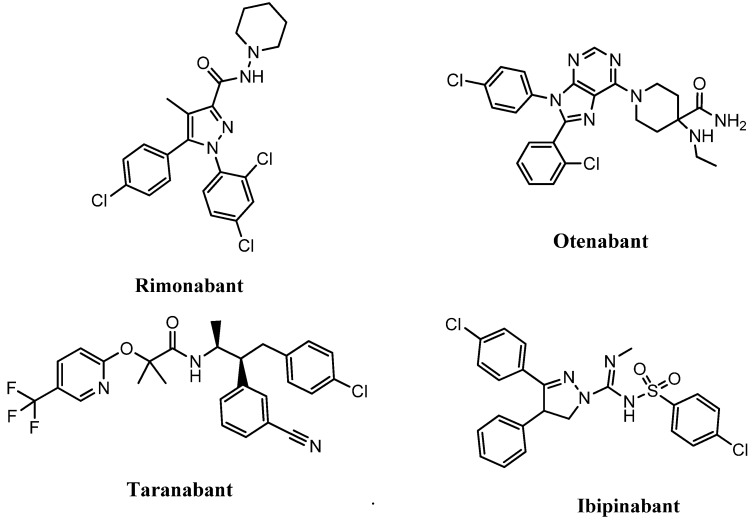
The type-1 cannabinoid receptor (CB1) antagonists: Rimonabant, taranabant, otenabant and ibipinabant.

Type 1 endocannabinoid receptors and endogenous cannabinoids are both involved in the management of appetite stimuli and food intake in the central region of the hypothalamus [1]. In the autonomic nervous system, the hypothalamus is deputed to the control of metabolism’s functions, fat storage and weight. The hypothalamus constantly receives feedback signals depending on metabolic requests in terms of energy needs to balance energy demand in order to regulate the food intake [2]. This process is modulated by two inputs: (i) The short and medium-term hunger signals of orexigenic input and satiety as anorexygenic inputs modify feeding behavior, and (ii) based on adiposity, the amount of the energy stored as fats produces a neurohormone called leptin [3]. The endogenous cannabinoid system (ECS) has been related to the modulation of the reward mechanism, which is a well-defined neural network deputed to motivation, desire, craving, learning, and positive emotions associated with pleasure, such as joy in specific mesencephalic zones mostly related to reinforcing mechanisms. In rats, orexigenic stimuli have been observed after the administration of endogenous cannabinoids in the hypothalamus, which indicates the role of the ECS located in the limbic forebrain zone in the activation of the motivational feeding mechanism [4]. According to some other works, endocannabinoids, namely anandamide and arachidonic ester of glycerol (2-AG), stimulate the type-1 cannabinoid receptor (CB1) and simultaneously induce the reduction of energy consumption, activating food grabbing behavior. In laboratory animals, cannabinoid agonists have demonstrated orexigenic effects; the administration of Δ^9^-tetrahydrocannabinol (THC) or anandamide into the hypothalamus also induced food intake [5]. Indeed, CB1^−/−^ mice or animals previously injected with CB1 antagonists have been found to assume an anorexigenic behavior [6]; when administered to drug naive animals, CB1 antagonists such as 5-(4-chlorophenyl)-1-(2,4-dichloro-phenyl)-4-methyl-*N*-(piperidin-1-yl)-1*H*-pyrazole-3-carboxamide (rimonabant) are able to evoke anorexigenic effects in rodents [7]. Furthermore, the ECS might be involved in the secretion of neuropeptides which play a pivotal role in the feeding mechanism at the hypothalamus, such as dynorphin A, endomorphin-2, met-enkephalin and leu-enkephalin. These endogenous peptides increase food grabbing behavior through an interaction with opioid receptors. Through motivational and rewarding mechanisms, the opioid system may activate feeding intake alone or by cross-talking with other systems present at the striatum level [8,9]. Other interactions have been found between the endocannabinoid and the orexin systems. Sub-effective doses of rimonabant are able to prevent the orexigenic effect of orexin A by increasing food intake in starving conditions in the short term. The physical dimerization between CB1 and orexin A receptors could be due to their co-expression in several areas of the hypothalamus, as observed in experiments with co-transfected cells [10,11]. Recently, some bioactive substances such as sibutramine and rimonabant have been used to reduce body weight due to their modulation of several neuroendocrine mediators, despite their serious side effects [12]. Sibutramine is one of the most frequently hidden drug ingredients in products claimed to be food supplements. The Food and Drug Administration (FDA) has recently highlighted an adulteration of a supplement-like weight loss, which was marketed in the United States by Abbott Laboratories as a prescription weight loss aid under the brand name Meridia [13]. The drug was retrieved from the market due to increased blood pressure and heart rate, which are risk factors in patients with coronary heart disease (CHD), arrhythmia and stroke. An early clinical trial was done on the CB1 antagonist rimonabant, during which the patients were treated with 20 mg of this drug. The results showed an amelioration of metabolic and cardiac parameters related to type 2 diabetes and heart diseases [14], a decrease of triglyceride, and an increase in high-density lipoprotein (HDL) cholesterol levels. Other effects have been also observed, including a reduction of waist circumference and body fat, as well as an improvement of tolerance to glucose and blood pressure [15]. However, in 2008, Sanofi-Aventis and Pfizer suspended research activity on rimonabant and its related chemical analogues with similar pharmacological profiles due to their serious side effects on the central nervous system [16]. Thus, the development of CB1 antagonists that act selectively in peripheral tissues represents an important task to overcome these drawbacks in the treatment of obesity or related diseases (Figure 1) [17].

**Scheme 2:**
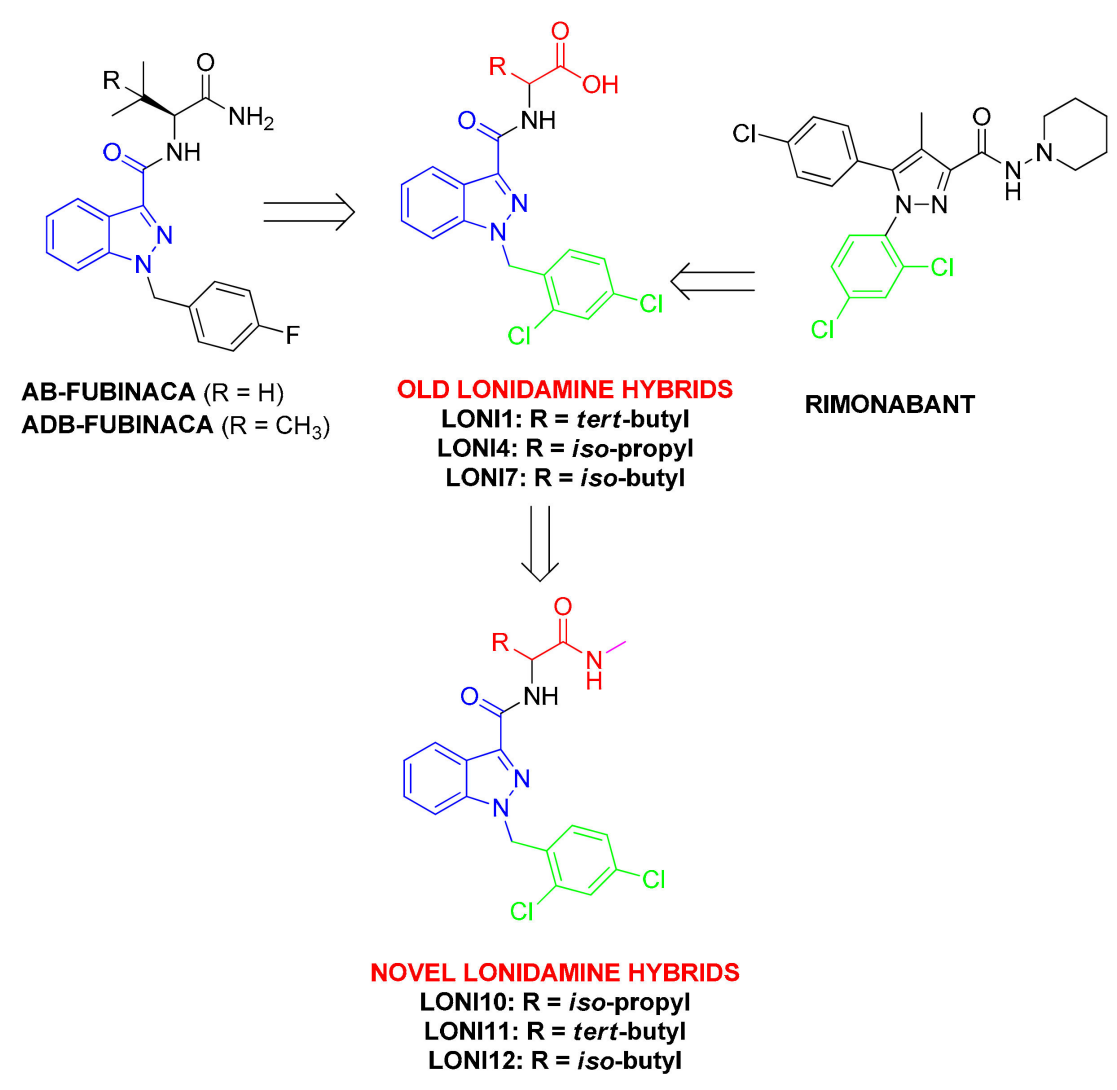
Chemical modifications applied to lonidamine structure in the design of novel compounds LONI10-12.

Taranabant and otenabant have different structures than rimonabant, but they share similar side effects, which resulted in the early end of their clinical studies [17,18]. In this context, the concept of “biased signaling” provides an exciting new direction for developing therapeutics with less adverse effects. The CB1 and CB2 receptors interact with several pathways by different signaling cascades; thus, they possibly bias other effects by the fine-tuning of the receptors conformations, allowing them to address specific symptoms or pathologies without side effects [19,20]. Given their importance as potential therapeutics for overweight and other dysmetabolisms, psychiatric symptoms, liver disfunction, and addiction to nicotine [21,22,23,24,25,26,27], our research group developed hybrids of rimonabant and the *N*-(2*S*)-substitutes of 1-[(4-fluorophenyl)methyl]indazole-3-carboxamide with 1-amino-3-methyl-1-oxobutane (AB-Fubinaca), 1-amino-3,3-dimethyl-1-oxobutane (ADB-Fubinaca), and 3-methylbutanoate (AMB-Fubinaca), endowed with potent agonistic activity towards cannabinoid receptors CB1 and CB2 (Scheme 2). Synthetic cannabinoids (SCs) AB-FUBINACA and ADB-FUBINACA (Scheme 2) have been identified in illicit drugs in the Japanese market. The administration of Fubinaca family compounds is associated with serious side effects, neurotoxicity and cardiotoxicity. They exert potent psychotropic adverse effects, including delirium and drug seizures, which have reportedly led, on some occasions, to hospitalization and death. Coronary arterial thrombosis in combination with SC use was ascertained as one of the major causes of death [28,29,30]. Numerous cases of fatal intoxication have led to the withdrawal of these products from the market in the US, Germany and China [31,32]. 

Our novel compounds have high CB1 receptor affinity and selectivity with different biological activity depending on the *C*-terminal substitution and amino acid residues; given their close structural similarity with rimonabant, we hypothesized a similar biological activity in vivo in the feeding behavior modulation. It was found that compounds containing the *C*-terminal methyl ester of *tert*-Leu (S)-methyl 2-(1-(2,4-dichlorobenzyl)-1H-indazole-3-carboxamido)-3,3-dimethylbutanoate (LONI2) and Val as the free acid ((S)-2-(1-(2,4-dichlorobenzyl)-1H-indazole-3-carboxamido)-3-methylbutanoic acid (LONI4)) are able to decrease food intake at 10 mg/Kg, acting as inverse agonists/antagonists at the CB1. Thus, it has been supposed that the *C*-terminal methyl ester group could be easily cleavable in vivo to give the free *C*-terminal acid derivative.

With the aim to stabilize the agonist activity of this series of compounds [33], we designed *N*-methyl amide derivatives of 1-(2,4-dichlorobenzyl)-1H-indazole-3-carboxylic acid (lonidamine) joined to Leu, Val and *tert*-Leu amino acids. In this study, we were able to demonstrate that compound (S)-1-(2,4-dichlorobenzyl)-*N*-(3,3-dimethyl-1-(methylamino)-1-oxobutan-2-yl)-1H-indazole-3-carboxamide (LONI11) is a full agonist of the CB1 receptor endowed with an orexant effect, while the previously published *C*-terminal methyl ester and the compounds of lonidamine with *tert*-Leu (LONI1) and Val (LONI4) as free acids showed an anorexic profile in vivo. Furthermore, several antinociception models were also studied to characterize the analgesic potential of this novel compound (LONI11).

## 2. Materials and Methods

### 2.1. Chemistry

Lonidamine, solvents, reagents and amino acids Boc-*tert*-Leu-OH, Boc-Val-OH, and Boc-Leu-OH, are commercially available and were acquired from Sigma-Aldrich (Milano, Italy). The intermediate compounds LONI1-4,7 were synthetized, as previously published by Stefanucci et al. [33]. The structures of the intermediates and the final compounds were confirmed by ^1^H-NMR and ^13^C-NMR spectra recorded on a 300 MHz Varian Inova spectrometer (Varian Inc., Palo Alto, CA). Chemical shifts were reported in parts per million (δ) downfield from the internal standard tetramethylsilane (Me_4_Si). The purity of each final product was established by analytical reverse phase-high performance liquid chromatography (RP-HPLC) (C18-bonded 4.6 × 150 mm) at a flow rate of 1 mL/min by using (as an eluent) a gradient of H_2_O/ACN 0.1% TFA ranging from 10% ACN to 90% ACN for 30 min; it was found to be >95% (see Supplementary Materials). UV detection at 254 nm was chosen for analytical HPLC. Mass spectra were performed on an LCQ (Finnigan–Mat) ion trap mass spectrometer (San Jose, CA, USA) equipped with an electrospray ionization (Supplementary Materials) source. The capillary temperature was set at 300 °C, and the spray voltage was set at 3.5 kV. The fluid was nebulized using nitrogen as both the sheath gas and the auxiliary gas [34,35,36].

#### General Procedure for the N-Methyl Amide Formation

HOBt anhydrous (1.1 eq.) in DMF (3 mL), EDC^.^HCl (1.1 eq.) and NMM (1 eq.) were added To a stirred solution of lonidamine-amino acid compound (200 mg) at 0 °C; this was followed by the addition of a solution of methylamine 40% in water (2 eq.) and NMM (2 eq.) in DMF (3 mL). After 10 min at 0 °C, the reaction was stirred at room temperature (r.t.) overnight. Later, the reaction mixture was evaporated to dryness, and the residue taken up in ethyl acetate (EtOAc). The organic phase was washed with 5% citric acid, NaHCO_3_ saturated solution (s.s.) and NaCl s.s., dried and evaporated in a high vacuum. The crude compound was triturated in Et_2_O two times to give the desired white solid product. The characterization of the final LONI10-12 compound is reported in the Supplementary Materials. 

(S)-2-(1-(2,4-dichlorobenzyl)-1H-indazole-3-carboxamido)-3,3-dimethylbutanoic acid (LONI1). Boc-*tert*-Leu-OH was coupled to lonidamine according to Stefanucci et al. [33].

(S)-methyl-2-(1-(2,4-dichlorobenzyl)-1H-indazole-3-carboxamido)-3,3-dimethylbutanoate (LONI2). Boc-*tert*-Leu-OH was transformed in its methyl ester derivative and coupled to lonidamine according to Stefanucci et al. [33].

(S)-*N*-(1-amino-3,3-dimethyl-1-oxobutan-2-yl)-1-(2,4-dichlorobenzyl)-1H-indazole-3-carboxamide (LONI3). Boc-L-*tert*-Leu-OH was converted into the amide derivative and coupled to lonidamine according to Stefanucci et al. [33].

(S)-2-(1-(2,4-dichlorobenzyl)-1H-indazole-3-carboxamido)-3-methylbutanoic acid (LONI4). Boc-Val-OH was coupled to lonidamine according to Stefanucci et al. [33].

(S)-1-(2,4-dichlorobenzyl)-*N*-(3-methyl-1-(methylamino)-1-oxobutan-2-yl)-1H-indazole-3-carboxamide (LONI10). The LONI4 compound was transformed in the *N*-methyl amide derivative LONI 10 following the general procedure. The desired compound was obtained in a 96% yield after reaction work up. 

(S)-1-(2,4-dichlorobenzyl)-*N*-(3,3-dimethyl-1-(methylamino)-1-oxobutan-2-yl)-1H-indazole-3-carboxamide (LONI11). The LONI1 compound was transformed in the *N*-methyl amide derivative LONI 11 following the general procedure. The desired compound was obtained in a 97% yield after reaction work up. 

(S)-1-(2,4-dichlorobenzyl)-*N*-(4-methyl-1-(methylamino)-1-oxopentan-2-yl)-1H-indazole-3-carboxamide (LONI12). The LONI7 compound was transformed in the *N*-methyl amide derivative LONI12 following the general procedure. The desired compound was obtained in a quantitative yield after reaction work up. 

### 2.2. In Vitro Biological Assays 

#### 2.2.1. Preparation of Brain Membrane Homogenates

Wistar rats were locally bred and handled according to the EU Directive 2010/63/EU and to the Regulations on Animal Protection (40/2013. (II. 14.) Korm. r.) of Hungary. Crude membrane fractions were prepared from the brain. Brains were quickly removed from the euthanized rats and directly put in an ice-cold 50 mM Tris–HCl buffer (pH 7.4). The collected tissue was then homogenized in 30 volumes (*v/w*) of an ice-cold buffer with a Braun Teflon-glass homogenizer at the highest rpm. The homogenate was centrifuged at 20,000× *g* for 25 min, and the resulting pellet was suspended in the same volume of a cold buffer followed by incubation at 37 °C for 30 min to remove endogenous ligands. Centrifugation was then repeated. The final pellets were taken up in five volumes of a 50 mM Tris–HCl (pH 7.4) buffer containing 0.32 M sucrose and stored at −80 °C. Prior to the experiment, aliquots were thawed and centrifuged at 20,000× *g* for 25 min and then they were resuspended in 50 mM Tris–HCl (pH 7.4), homogenized with a Douncer, followed by the determination of the protein concentration by the method of Bradford. The membrane suspensions were immediately used either in radioligand binding experiments or in [^35^S]GTPγS functional assays.

#### 2.2.2. Radioligand Competition Binding Assay

Binding experiments were performed at 30 °C for 60 min in a 50 mM Tris–HCl binding buffer (pH 7.4) containing 2.5 mM of EGTA, 5 mM of MgCl_2_ and 0.5 mg/mL of fatty acid-free BSA in plastic tubes in a total assay volume of 1 mL that contained 0.3–0.5 mg/mL of a membrane protein [33,37]. Competition binding experiments were carried out by incubating rat brain membranes with 5 nM of [^3^H]WIN55212-2 (K_d_: 10.1 nM) in the presence of increasing concentrations (10^−11^–10^−5^ M) of various competing unlabeled ligands. Non-specific binding was determined in the presence of 10 µM of WIN 55212-2. The incubation was terminated by diluting the samples with an ice-cold wash buffer (50 mM of Tris–HCl, 2.5 mM of EGTA, 5 mM of MgCl_2_, 0.5% fatty acid free BSA, pH 7.4), followed by repeated washing and rapid filtration through Whatman GF/B glass fiber filters (Whatman Ltd., Maidstone, UK) presoaked with 0.1% polyethyleneimine (30 min before the filtration). Filtration was performed with a 24-well Brandel Cell Harvester (Gaithersburg, MD, USA). Filters were air-dried and immersed into Ultima Gold MV scintillation cocktail, and then radioactivity was measured with a TRI-CARB 2100TR liquid scintillation analyzer (Packard, Perkin Elmer, Waltham, MA, USA).

#### 2.2.3. Ligand Stimulated [^35^S]GTPγS Binding Assay

Rat brain membranes (30 µg protein/tube), prepared as described above, were incubated with 0.05 nM of [^35^S]GTPγS (PerkinElmer) and 10^−10^–10^−5^ M unlabeled ligands in the presence of 30 µM of GDP, 100 mM of NaCl, 3 mM of MgCl_2_ and 1 mM of EGTA in a 50 mM Tris–HCl buffer (pH 7.4) for 60 min at 30 °C. Basal [^35^S]GTPγS binding was measured in the absence of ligands and set as 100%. Nonspecific binding was determined by the addition of 10 µM unlabeled GTPγS and subtracted from total binding. Incubation, filtration and radioactivity measurements of the samples were carried out as described above.

#### 2.2.4. Data Analysis

The results of the competition binding studies are reported as means ±S.E.M. of at least three independent experiments each performed in duplicate. In competition binding studies, the inhibitory constants (K_i_) were calculated from the inflection points of the displacement curves using non-linear least-square curve fitting and the Cheng–Prusoff equation, K_i_= EC_50_/(1 + [ligand]/K_d_).

In [^35^S]GTPγS binding studies, data were expressed as the percentage stimulation of the specific [^35^S]GTPγS binding over the basal activity and are given as means ±S.E.M. Each experiment was performed in triplicate and analyzed with sigmoid dose–response curve fitting to obtain potency (EC_50_) and efficacy (E_max_) values. All data and curves were analyzed by GraphPad Prism 5.0 (San Diego, CA, USA).

### 2.3. In Vivo Biological Assays

#### 2.3.1. Animals

The international and national law and policies approved by Italian Ministry of Health were used to comply with all animal care and experimental procedures. Animal studies were advised in compliance with the ARRIVE guidelines and with the recommendations made by EU Directive 2010/63/EU for animal experiments and the Basel declaration including the 3Rs concept [38,39]. CD-1 male mice (10–14 weeks of age, 25–30 g of weight) were bought from Charles River (Milan, Italy). Shortly after their arrival and for at least one week, they were kept in an animal care facility under controlled standard conditions of temperature (21 ± 1 °C), light (from 7:00 AM to 7:00 PM), and relative humidity (60 ± 10%). Access to drinking water and food was assured. All procedures were performed to decrease the number of animals used (n = 6 per group) and their distress.

#### 2.3.2. Feeding Test

The test was carried out as previously described [40]. At 24 h before the start of a feeding test, all food was removed from the home cages of mice to be tested. The next day and at least 1 h before the feeding test began, the mice were transported to the laboratory. On test days, the animals were placed in the home cages for 30 min of drug assimilation, during which food was not available. Then compounds were intraperitoneally administered (10 mg/kg). Mice were transferred into transparent and individual plastic cages with thick white paper lining the bottom and access to a pre-measured amount of their regular lab chow (2 gr) for the 1-h test. At the end of 1 h, mice were repositioned into their home cage. The amount of food left in the trial cage, including crumbs, was measured, and the amount consumed was calculated. Feeding trials normally happened on Tuesdays and Fridays between 12:00 and 14:00 h. 

#### 2.3.3. Tail Flick Test

The tail flick test was used to determinate antinociceptive responses [41]. Tail flick latency (Ugo Basile, Varese, Italy) consists of an infrared radiant light source (100 W, 15 V bulb) targeted on a photocell utilizing an aluminum parabolic mirror. During the trials, the mice were gently hand restrained with a glove. Radiant heat was targeted 5–6 cm from the tip of the tail, and the latency (s) of the tail withdrawal recorded. The measurement was disconnected if the latency crossed the cutoff time. A cutoff time of 15 s was imposed, and data were expressed as time course of the percentage of maximum effect (% MPE) = (post drug latency/baseline latency)/(cutoff time baseline latency) × 100. In all experiments, the baseline was calculated as the mean of three readings recorded before testing at intervals of 10 min, and the time course of latency was determined 10–120 min after compound treatment. Compounds were freshly diluted in saline 0.1% *v*/*v* DMSO and were injected at 10 µg/10 µL for intracerebroventricular (i.c.v.) administrations, as previously reported [42,43].

#### 2.3.4. Formalin Test 

The method utilized was comparable to the one previously described by Pieretti et al. [44]. Mice were located to adapt into the transparent cages individually (30 × 14 × 12 cm) for at least an hour before testing. They were injected with 20 μL of a 1% solution of formalin in saline. Then, the compounds were administered subcutaneously in the dorsal surface of the right hind paw of the mouse using a microsyringe with a 27-gauge needle for 15 min before. Compounds were prepared by freshly diluting saline containing 0.9% NaCl in the ratio DMSO:saline 1:3 (*v/v*). Then, these solutions were injected for subcutaneous (s.c.) administrations in doses of 30–100 µg/20 µL. The total time the animal spent licking or biting its paw was calculated. 

#### 2.3.5. Edema Induced by Zymosan

In this test, 100 µg of the compounds were administered subcutaneously in a volume of 20 µL in the dorsal surface of mice hind paw; this was done 15 min before a subcutaneous injection (20 μL/paw) of zymosan A (2.5% *w/v* in saline) into the same paw. Then, paw edema was calculated as formerly described [45]. The percentage difference between the paw volume at each time point and the basal paw volume was used as an index of the increase in paw volume. Paw volume was quantified using a hydroplethysmometer modified for small volumes (Ugo Basile, Varese, Italy) three times before the injections and at 1, 2, 3, 4 and 24 h thereafter. 

#### 2.3.6. Zymosan-Induced Hyperalgesia

The compounds (100 µg) were administered subcutaneously in a volume of 20 µL in the dorsal surface of mice hind paw; this was done 15 min before a subcutaneous injection (20 μL/paw) of zymosan A (2.5% *w/v* in saline) into the same paw before the measurement of hyperalgesia [45]. The sensitivity to a noxious heat stimulus was measured by the plantar test (Ugo Basile, Italy), in order to evaluate thermal hyperalgesia after the zymosan-induced inflammation of the mouse hind paw. Mice were allocated in clear plastic boxes with a glass floor and acclimatized for at least 1 h in a temperature-controlled (21 °C) experimental room for three consecutive days prior to testing. Furthermore, on the test day, the animals were acclimatized to the experimental room 1 h before paw withdrawal latency (PWL) was calculated. Using a timer and in term of seconds, yhe paw withdrawal latency was measured automatically after placing the mouse footpad in contact with a radiant heat source. A timer initiated automatically when the heat source was activated, and a photocell stopped the timer when the mouse withdrew its hind paw. An intensity of 30 and a cut-off time of 15 s were used for the heat source on the plantar apparatus to avoid tissue damage. Animals were first tested to define their baseline PWL in terms of seconds against 1, 2, 3, 4, 5 and 24 h after zymosan A injection.

#### 2.3.7. Data Analysis and Statistics

The mean ±S.E.M. was used to explain the results obtained. Statistically significant differences between groups were measured with an analysis of variance (ANOVA) followed by Tukey’s post-hoc comparisons or the Mann–Whitney test when the comparison was restricted to two groups. GraphPad Prism 6.0 software (San Diego, CA, USA) was used to analyze the data. Data were considered statistically significant when a value of *p* < 0.05 was performed. The data and statistical analyses respected the recommendations on experimental design and analysis [46]. 

### 2.4. In Silico Experiments

#### 2.4.1. Receptor Preparation

The crystal structure of the human CB1 receptor co-crystallized with an MDMB-Fubinaca agonist was downloaded from the RSCB database (pdb id: 6N4B) [47]. The raw file was prepared for the docking experiment by the PrepWizard module of Maestro 10.2 [48]. Briefly, the missing chains were added automatically by Prime [49], and the protonation state was calculated by PropKa at pH = 7.4 [48]. Finally the receptor–ligand complex was minimized by OPLS-3 force field following a well-established protocol reported by our research group [42,50].

#### 2.4.2. Docking Grid Generation

The docking grid was generated by the Glide module of Maestro [51]. The grid was centered on the MDMB-Fubinaca ligand present in the crystal structure and extended to a space of 20 × 20 × 20 Angstrom. The generated grid was used for the docking experiments.

#### 2.4.3. Self-Docking and Validation Procedure

In order to validate the docking procedure, a self-docking experiment was conducted. The crystallographic ligand MDMB-Fubinaca was removed from the receptor, prepared, and minimized by the LigPrep module [52] of Maestro using EpiK at pH = 7.4 [48]. The software generated 32 minimized structures, and the best ranked structure was used for the self-docking procedure. The obtained ligand was submitted to a first round of docking by using Glide at Standard Precision (Glide-SP) accuracy. The best raked pose was subjected to a second round of docking by using Glide in eXtra Precision (Glide-XP) mode. Then, the RMSD of the best ranked pose was measured as 0.5 Å below the original crystallographic pose (see Figure S1). This procedure was applied to the docking experiments of the novel compounds.

#### 2.4.4. Ligands Preparation

LONI4 and LONI11 were drawn by the 2D editor embedded in Maestro and prepared by the LigPrep module following the same procedure applied to MDMB-Fubinaca. Then, the minimized structures were submitted to the docking experiments without further modifications.

#### 2.4.5. Ligand Docking Experiments

The prepared molecules LONI4 and LONI11 were docked to the CB1 receptor. The first round of docking was performed by Glide in Standard Precision mode. The best pose generated from the first step was then submitted to the second round of docking by using Glide in eXtra Precision accuracy. The best ranked poses are depicted in Figure S2 as a bi-dimensional interaction diagram (see Supplementary Materials).

#### 2.4.6. Molecular Dynamic

The LONI4-CB1, LONI11-CB1 and MDMB-Fubinaca-CB1 complexes obtained from self-docking were also submitted to molecular dynamic (MD) experiments by the Desmond module embedded in Maestro 12.0 [53]. The MD simulation system was composed of the receptor–ligand complex embedded into a dipalmitoylphosphatidylcholine (DPPC) membrane-bilayer surrounded by water (Figure S3, see Supplementary Materials). Firstly, each complex was positioned in the membrane bilayer of DPPC lipids and then inserted into a water box to simulate a model of cellular bilayer system. The water box had a minimum size as to contain the receptor complex embedded in the membrane, ensuring a distance of 10 Å from the edge of the box and the protein. In order to neutralize the system, 0.15 M of NaCl was added. The OPLS-3 force field was used for all the experiments, and the TIP4P model was used for the water [54]. The system was minimized up to 2000 steps, holding all the protein and ligand atoms. Then, the minimized system was subjected to MD simulations, using the NPT ensemble and periodic boundary conditions for 20 ns. The Martyna–Tobias–Klein algorithm [55] was used to keep the pressure of the system at 1.01 bar by using the isotropic coupling method. The Nose–Hoover thermostat was applied to control the temperature at 310K [56]. The trajectories and other parameters were saved every 20 and 1.2 ps, respectively, to return 1000 frames. The simulation analysis was done by the simulation interactive diagram (Supplementary Materials, to visualize the RMSD fluctuations of the ligand and the receptor, the hydrogen bonds stability, the water network formation and the overall stability of the secondary structure of the enzyme.

## 3. Results

**Scheme 3:**
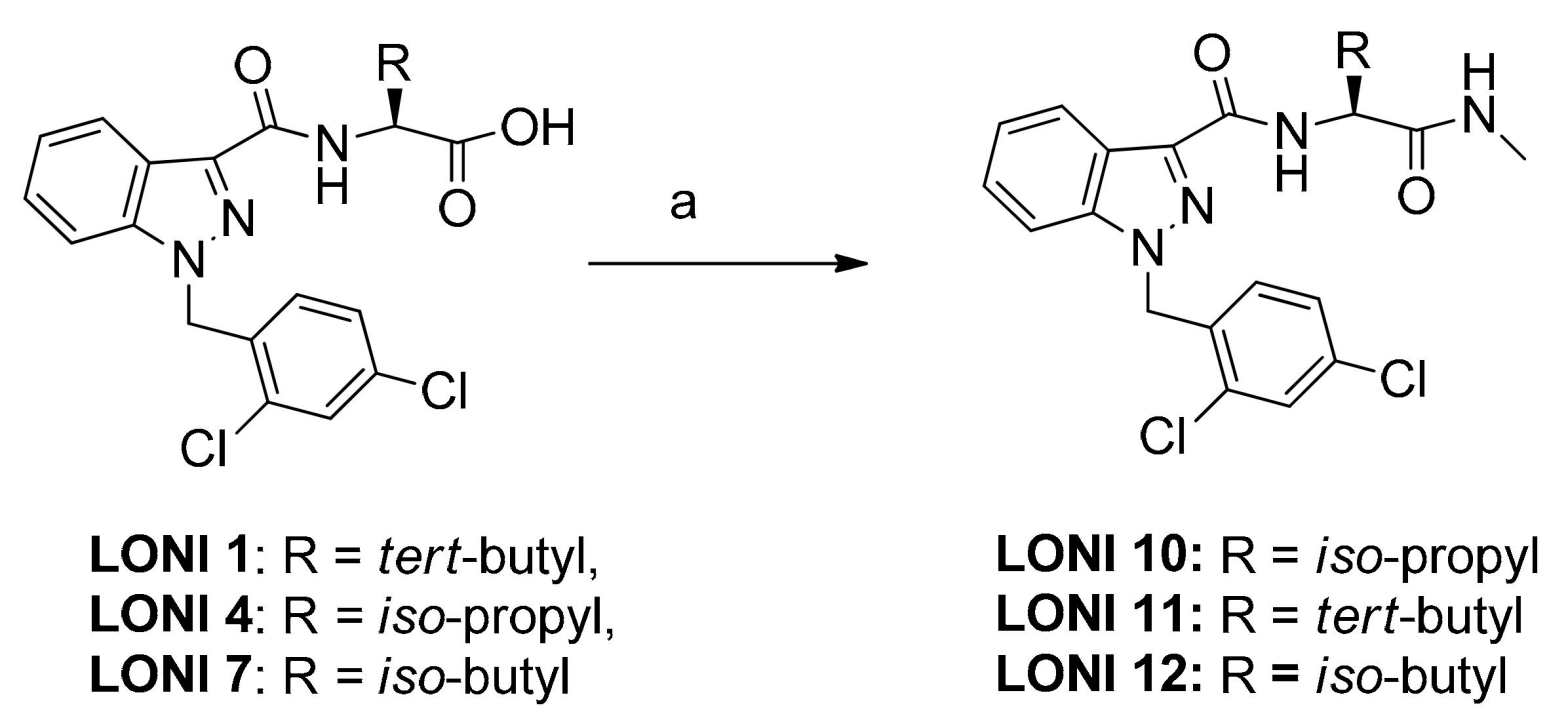
Reagents and Conditions: (**a**) EDC·HCl (1.1 eq.), HOBt an. (1.1. eq.), NMM (3 eq.), methylamine 40% in water (2 eq.), DMF (6 mL), r.t. 12 h (LONI10: 96% yield; LONI11: 97% yield; LONI12: Quantitative).

The LONI1-4,7 compounds were prepared by a well-established procedure previously reported by Stefanucci et al. [33]. The LONI10-12 novel chemical entities were efficiently recovered in excellent yields by standard solution phase peptide synthesis using EDC/HOBt coupling reagents, NMM as a base, and a solution of methylamine 40% in water (Scheme 3). All the final compounds were triturated in diethyl ether two times and then characterized by low resolution mass spectroscopy (LRMS), ^1^H- and ^13^C-NMR; the purity of the final products was determined by analytical RP-HPLC and found to be >95% (see Supplementary Materials). 

**Figure 2:**
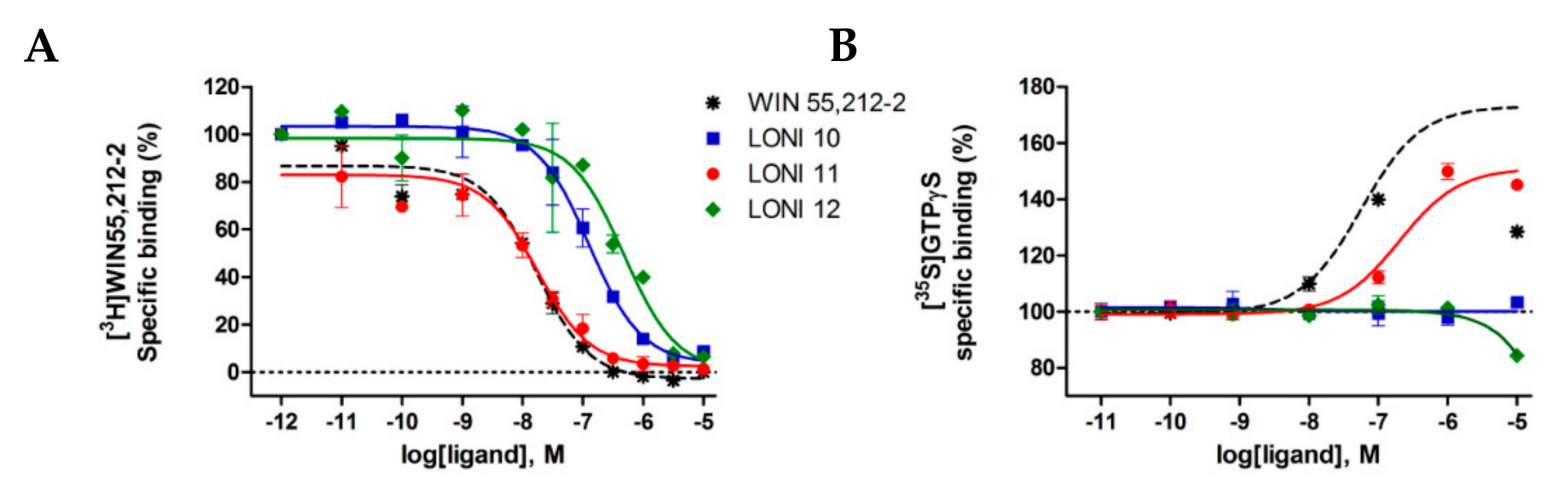
(**A**) CBs receptor binding affinity of LONI 10-12 in [^3^H]WIN55,212-2 competition binding assays to rat whole brain membrane homogenates. Figures represent the specific binding of the radioligand in percentage in the presence of increasing concentrations (10^−10^–10^−5^ M) of the indicated ligands. Data are expressed as percentage of mean specific binding ± S.E.M. (*n* ≥ 3). (**B**) G protein activation effects of the novel ligands in [^35^S]GTPγS binding assays in rat brain membrane homogenates. Figures represent the relative specific binding of [^35^S]GTPγS in the presence of increasing concentrations (10^−10^–10^−5^ M) of the indicated compounds. Data are expressed as percentage of mean specific binding ±S.E.M. (*n* ≥ 3).

The novel compounds were tested for their property to bind to cannabinoid receptors and for their ability to activate the G protein-coupled receptors (Figure 2). Efficacy (E_max_) and potency (EC_50_) of lonidamine-based compounds are reported in Table 1.

**Figure 3:**
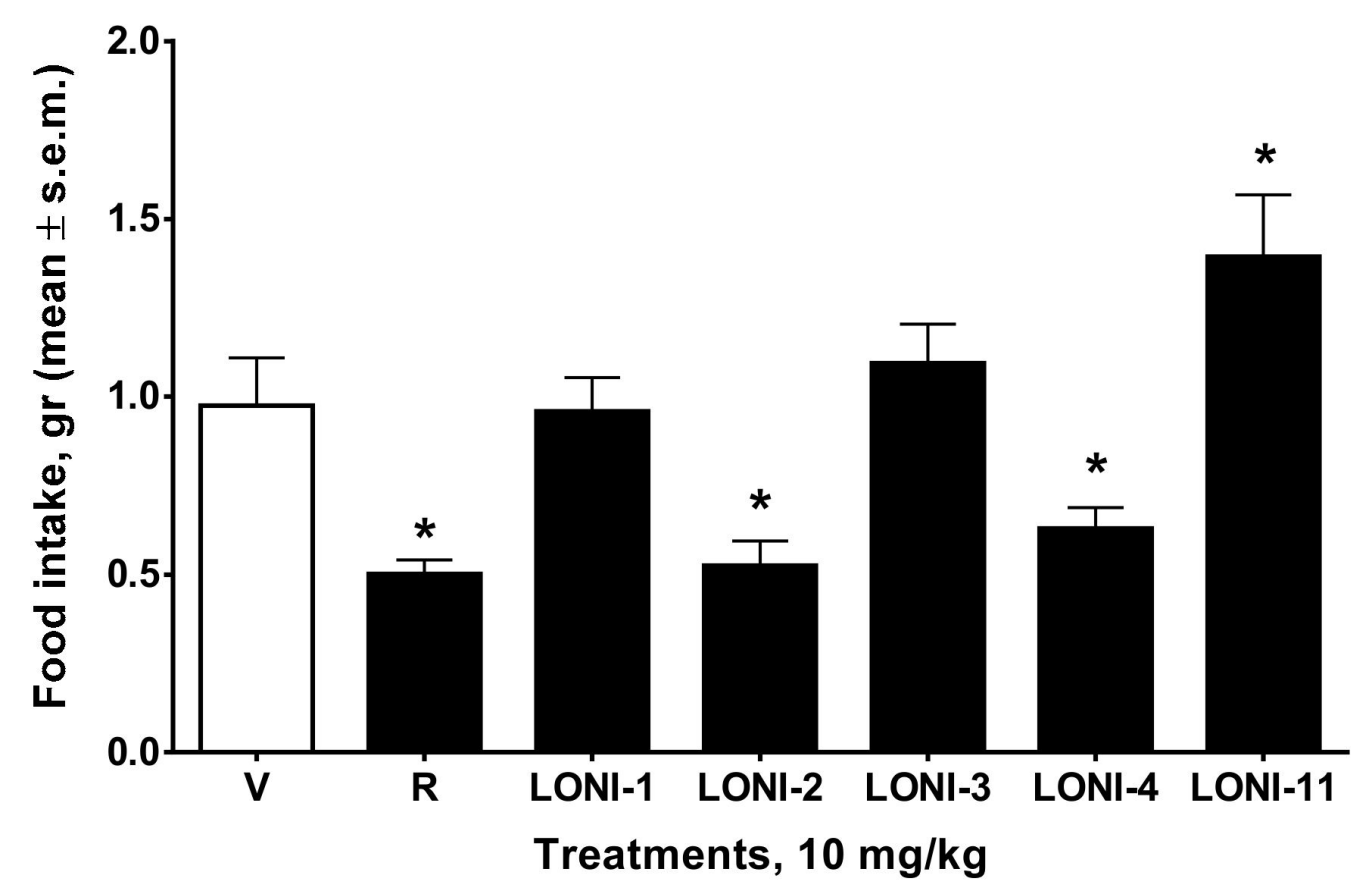
Activities of rimonabant, LONI1-4, LONI11 and vehicle (V, 1:1:18 mix of DMSO: Tween 80: sterile saline) on food intake. The compounds were injected intraperitoneally (10 mg/kg), and, after 30 min drug assimilation, mice were placed into individual cages and with access to a pre-defined amount of their regular lab chow (2 gr) for the 1-h test. Bars represent the mean ± s.e.m. of data from the same six mice at each dose for food intake. * is for *p* < 0.05 vs. V (vehicle-treated animals). N = 6.

The biological in vitro assay revealed that LONI11 was able to bind CB1 with a Ki value (11 nM) very close to that of WIN55,212 (10 nM), and it was also able to stimulate the GTP-binding protein with an E_max_ of 151% but with three-fold less potency than WIN55,212. The other compounds bind the CB1 with less affinity and show antagonist properties. These results suggest that LONI11 binds CB1 and stimulates the GTP coupled to the CB1 receptor, showing a biological profile very similar to that of the full CB1 agonist WIN55,212. Encouraged by these data, we planned to study the potential orexant effect of LONI 1 in the in vivo model of food intake in comparison to the previously described compounds LONI1-4 which exhibit agonist and partial agonist profiles (Figure 3) [33].

**Figure 4:**
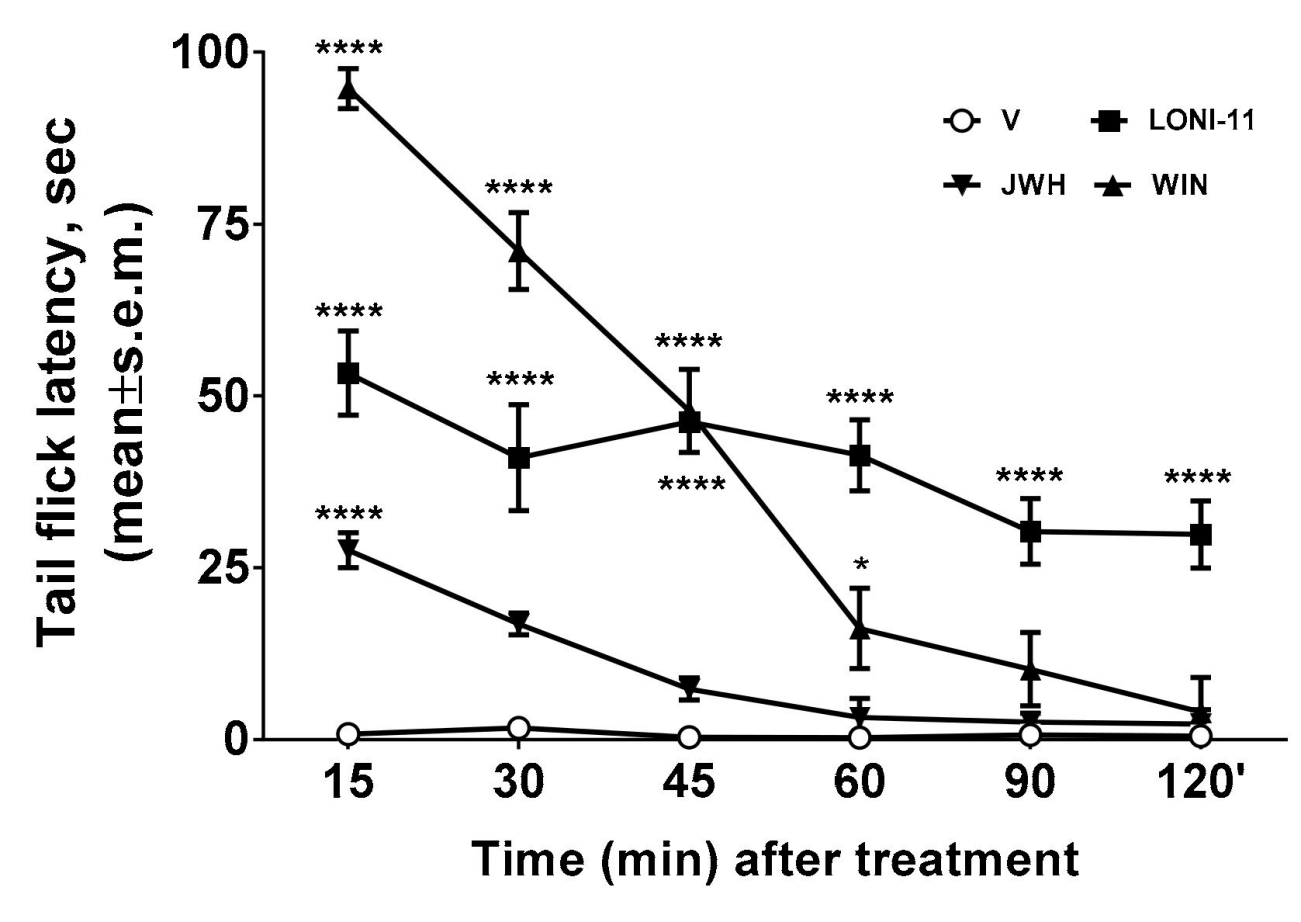
Antinociceptive effect of JWH-018 (JWH), WIN 55,212-2 (WIN), LONI11 and vehicle (V, saline 0.1% *v/v* DMSO) at the dose of 10 µg/10 µL after intracerebroventricular (i.c.v.) administration in the tail flick test. The antinociceptive activity is expressed as percentage of the maximum possible effect (%MPE ± s.e.m.). * is for *p* < 0.05 and **** is for *p* < 0.0001 vs. V (vehicle-treated animals). N = 6.

**Figure 5:**
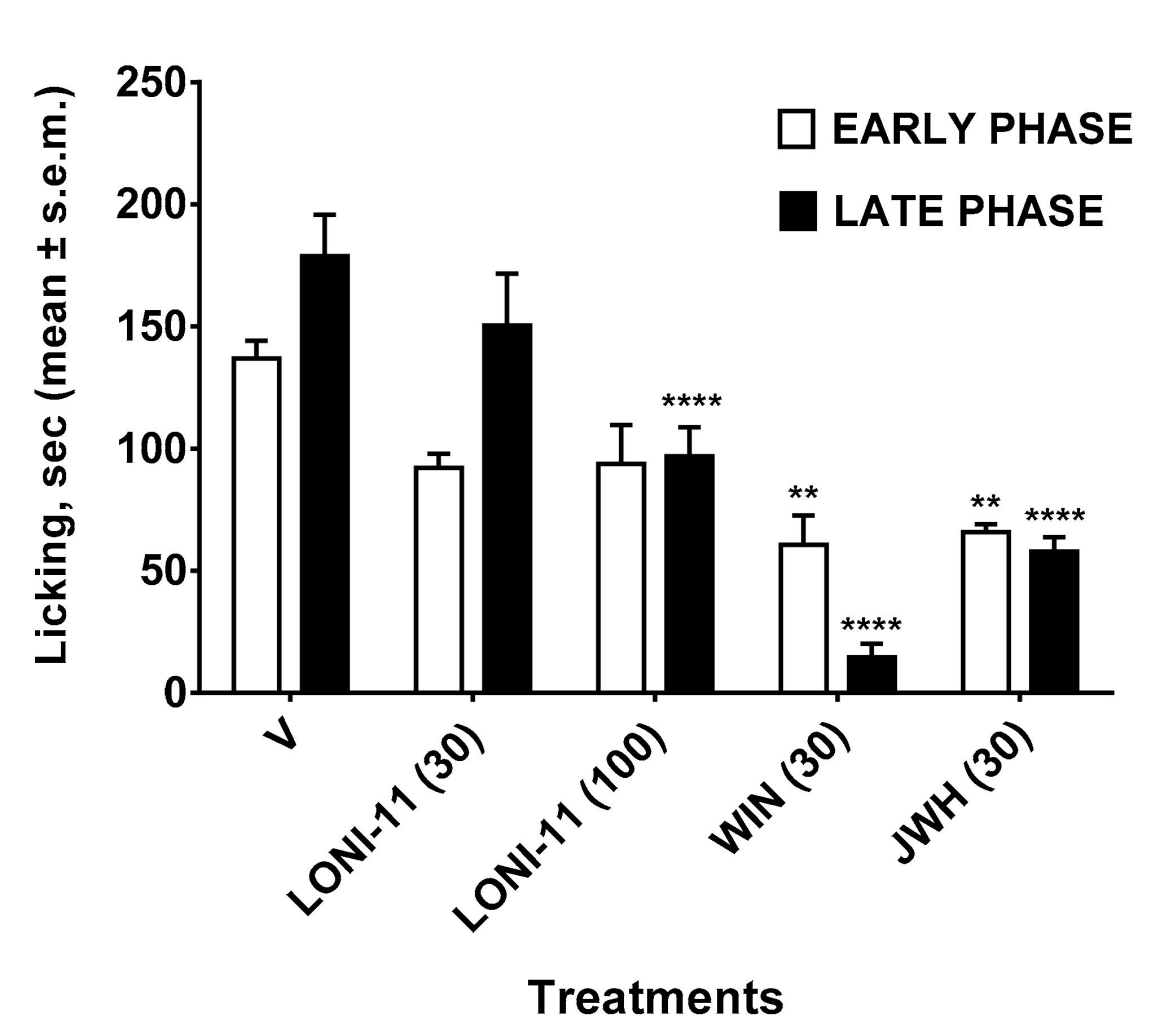
Effects induced in the early (white bars) and in the late (black bars) phase of the formalin test by vehicle (V, saline containing 0.9% NaCl in the ratio DMSO:saline 1:3 (*v/v*)), LONI11, WIN 55,212-2 and JWH-018 (JWH). The compounds were injected subcutaneously (s.c.) at a single dose (30–100 µg in 20 µL) 15 min before formalin injection (20 μL; s.c.). Early: Licking activity recorded from 0 to 10 min after formalin administration. Late: Licking activity recorded from 15 to 40 min after formalin administration. The results obtained are expressed as the mean ±S.E.M.; ** is for *p* < 0.01 and **** is for *p* < 0.0001 vs. V (vehicle-treated animals). N = 6.

The LONI1-4 reference compounds were ineffective for food intake at 3 mg/Kg per dose after i.p. administration, while LONI2 and 4 were able to reduce food intake at 10 mg/Kg per dose as the positive control rimonabant. Interestingly, as suggested by its agonist activity, the novel compound LONI11 increased the food intake at 10 mg/Kg per dose after i.p. administration, revealing a potential orexant effect. To better understand the pharmacological properties of this novel compound and to verify if LONI11 is able to exert a significative biological effect at the central level and periphery, we also performed tail flick and formalin tests in vivo. LONI11 at 10 μg/10 μL and 100 μg/20 μL produced a slight antinociceptive effect after i.c.v. and s.c. injections, respectively, in tail flick (Figure 4) and formalin tests (Figure 5).

**Figure 6:**
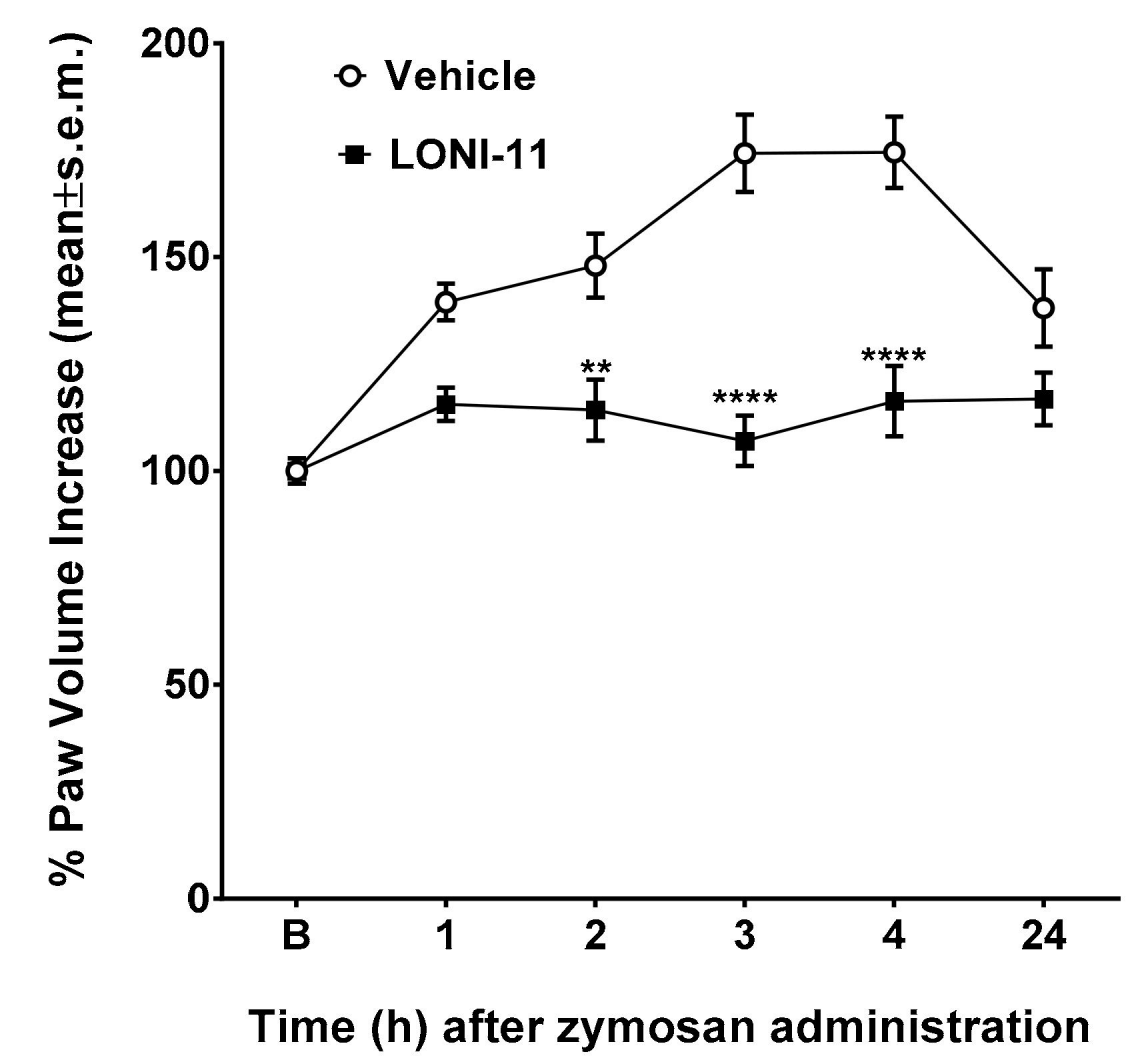
Effects of vehicle (V, saline containing 0.9% NaCl in the ratio DMSO:saline 1:3 (*v/v*)), and LONI11 on zymosan-induced edema. Zymosan (2.5% *w/v* in saline, 20 μL) was subcutaneously administered in the dorsal surface of the right hind paw. Drugs were administered subcutaneously in the dorsal surface of the right hind paw at the dose of 100 μg/20 μL, 15 min before zymosan. Paw volume was measured 1 h before zymosan and 1, 2, 3, 4 and 24 h thereafter. The paw volume increase was evaluated as the percentage between the paw volume at each time-point and the basal paw volume. The results obtained are expressed as the mean ±S.E.M.; ** is for *p* < 0.01 and **** is for *p* < 0.0001 vs. V. N = 6.

**Figure 7:**
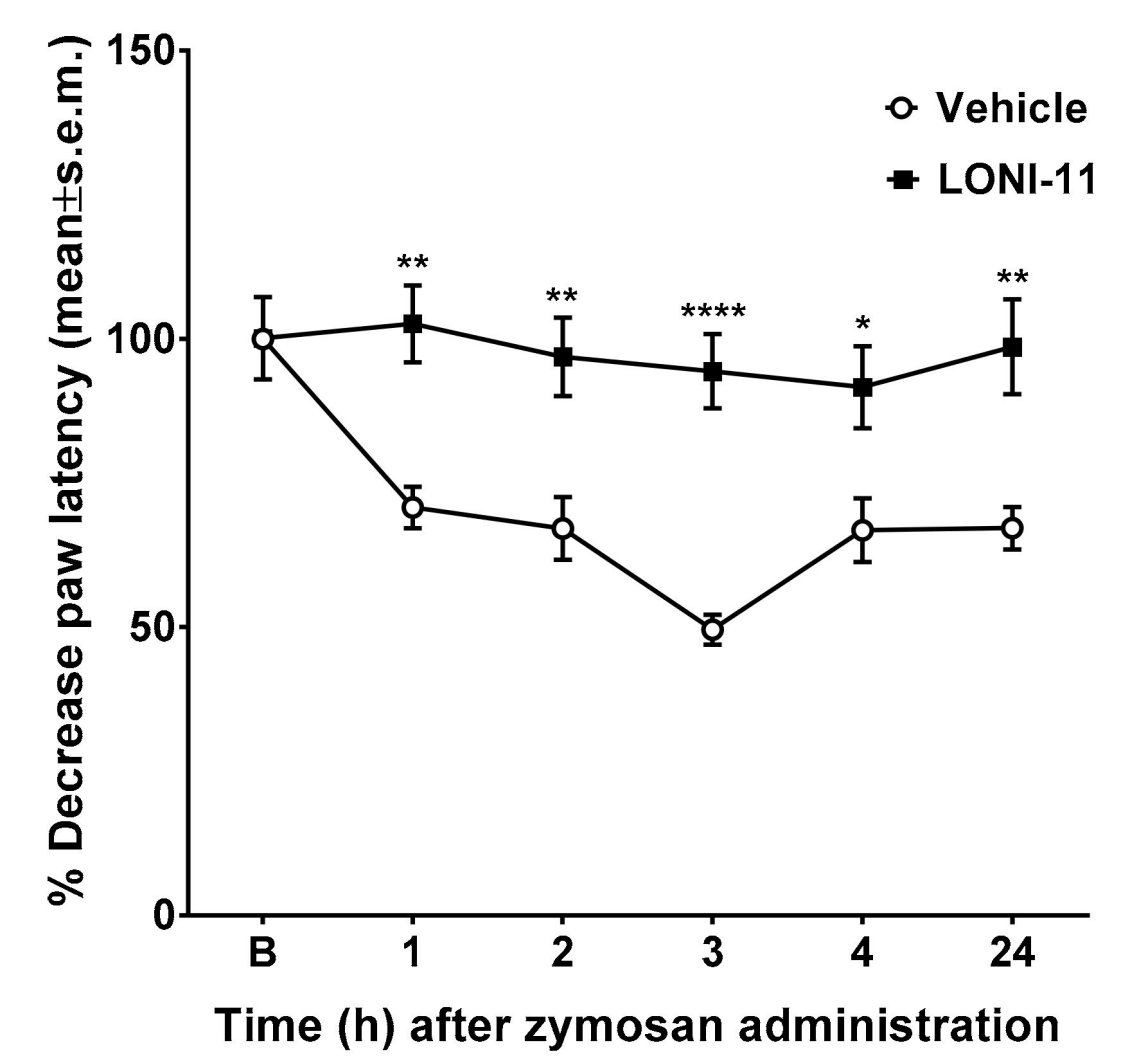
Effects of vehicle (V, saline containing 0.9% NaCl in the ratio DMSO:saline 1:3 (*v/v*)), and LONI11 on zymosan-induced paw hyperalgesia. Zymosan (2.5% *w/v* in saline, 20 μL) was subcutaneously administered in the dorsal surface of the right hind paw. Drugs were administered subcutaneously in the dorsal surface of the right hind paw at the dose of 100 μg/20 μL, 15 min before zymosan. Paw latency was measured 1 h before zymosan and 1, 2, 3, 4 and 24 h thereafter. The paw latency decrease was evaluated as the percentage between the paw latency at each time-point and the basal paw latency. The results obtained are expressed as the mean ±S.E.M.; * is for *p* < 0.05, ** is for *p* < 0.01 and **** is for *p* < 0.0001 vs. V. N = 6.

Zymosan-induced edema and iperalgesia assays were also performed to estimate the anti-inflammatory activity of our novel compound (Figure 6 and Figure 7). LONI11 at 100 μg/20 μL after s.c. route maintained the percent of paw volume at 100% for 24 h before zymosan administration (Figure 6), and it was also able to reduce the percent of paw latency to about 50% after 3 h following the s.c. administration at the same dose (Figure 7).

## 4. Discussion

Compounds LONI2 and LONI4, namely lonidamine-*tert*-LeuOCH_3_ and lonidamine-Val-OH, respectively, were found to inhibit food intake, consistent with an inverse agonism at CB1 receptors (Table 1) [33]. 

**Figure 8:**
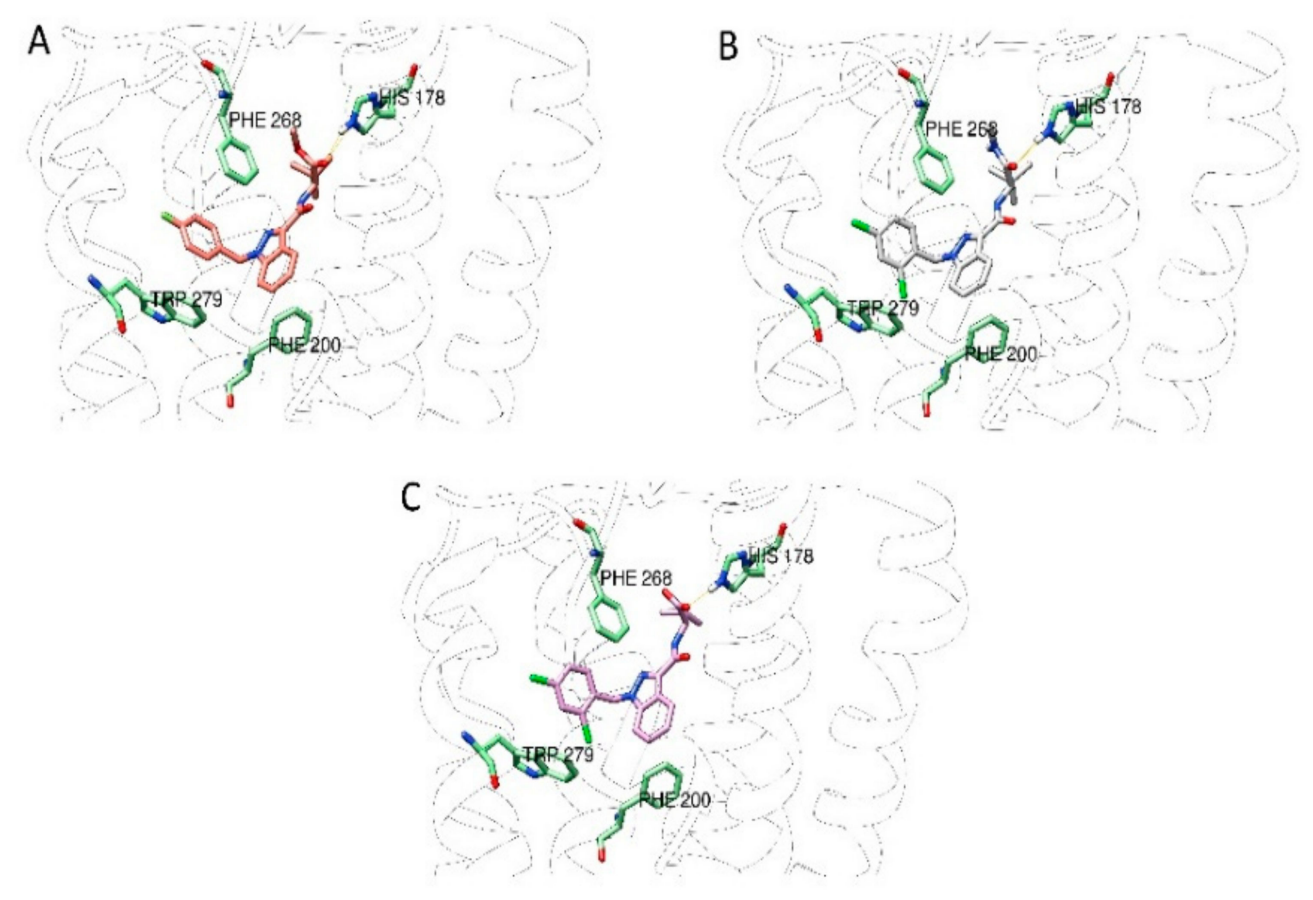
Best ranked pose of MDMB-Fubinaca (**A**), LONI11 (**B**) and LONI4 (**C**) docked to the CB1 receptor.

The *C*-terminal methyl ester LONI2, which has been shown to possess CB1 agonist activity in vitro, should be expected to enhance food intake in normal animals. LONI2 exerts an activity very similar to that of *C*-terminal acid (LONI4) and amide (LONI3) derivatives at 10 and 3 mg/Kg, respectively, probably due to its fast hydrolysis in vivo, which promptly converts LONI2 into LONI1 [57]. Then, we synthetized further analogues with the *N*-methyl amide terminus. Among them, LONI11 was the most potent agonist CB1 receptor. We tested this compound for food intake in vivo and revealed orexant activity, unlike the less chemically stable methyl ester analogue LONI2. We observed a significant orexant effect for LONI11 and an intense anorexant effect for LONI2 and LONI4 comparable to that of rimonabant (10 mg/Kg). Considering the well-established analgesic effect of the synthetic CB1 agonist THC on pain [58], the full agonist LONI11 was further tested to study the antinociceptive effect at the central and periphery levels. This compound was able to exert a good antinociceptive effect in tail flick and formalin tests in vivo following different administration routes. Furthermore, the subcutaneous administration of LONI11 at 100 mg/20 μL in zymosan induced edema and hyperalgesia rat models, decreased the percent of paw latency, and counteracted the percent of paw volume increase, suggesting potential activity at peripheral tissues. Though these interesting data prompted us to further investigate their effects upon chronic use, the toxicological study of these rimonabant/Fubinaca hybrids should be a primary issue. Documented side effects of Fubinaca series compounds are impaired driving, acute psychiatric distress, aggressiveness and polysubstance abuse with several SCs and alcohol [59,60,61,62,63,64]. These effects are also correlated to populations’ changes in consuming SCs, such as incarcerated persons, homeless, and their cultural heritage [65,66,67]. In order to better understand the molecular bases of LONI11 activity as an agonist and LONI4 activity as a partial inverse agonist, in silico docking experiments and molecular dynamic (MD) simulations were carried out. The best pose of LONI11 generated by Glide is depicted in Figure 8B. The key interactions observed in the crystal complex 6N4B were well represented in the docking pose of LONI11, namely the π–π interactions with Phe268, Trp279, Phe200 and the hydrogen bond formed with His178. The best docking pose of LONI11 was very close to that of the crystallographic ligand MDMB-Fubinaca (Figure 8A).

LONI4, which has been demonstrated to have partial inverse agonist activity, was also able to interact with the binding pocket of the CB1 receptor by forming the same key interactions found for LONI11 (see Figure 8B,C). The docking score of the best pose found was −11.7 Kcal/mol for LONI11, similar to that of MDMB-Fubinaca (−11.1 kCal/mol) and lower than that of LONI4 (−10.5 Kcal/mol). Thus, the difference of activity could have been only due to the *C*-terminal group. At physiological pH (7.4), the carboxylic moiety was largely deprotonated and the *C*-terminus of LONI4 beared a stable negative charge. However, the molecular docking experiments alone are not able to explain its different activity. Indeed, all the starting poses were similar each other, as highlighted in the docking experiments, bearing all the same interactions to His178, Phe200, Phe268 and Trp279. We further submitted the best pose of each compound to molecular dynamic simulation over 20 ns. The RMSD of the reference ligand MDMB-Fubinaca was found to be very low, fluctuating from 0.8 to 2.4 Å, which revealed a good and stable pose. The MD simulation showed significant differences from the starting pose: The Fubinaca analogue was able to form several hydrogen bonds to the water molecules present in the environment and to establish a water network with Ser383 and His178 (Figure S4; see Supplementary Materials). Thus, these two residues should have been be highly involved in the stabilization of Fubinaca ligand pose together with the previously observed Trp279, Phe200 and Phe268. This water network was also predicted by Kumar et al. and Michel et al. [47,68].

During the MD simulation, LONI11 maintained the π–π stack with the aromatic residues Phe268, Trp279 and Phe379 (Figure S4; see Supplementary Materials). However, the polar portion of the molecule largely lost the initial hydrogen bond to His178, and a water network between one water and the residue Ser383 was formed, as in the case of Fubinaca ligand. The presence of the free carboxylic group in LONI4 deeply influenced the stabilization of the starting docked pose during the MD simulation. Indeed, the RMSD plot of LONI4 ranged from 0.8 to 4.5 Å, demonstrating that the starting pose was scarcely stable (Figure S5; see Supplementary Materials). During the MD simulation, several novel interactions were formed, along with a strong hydration of the free carboxylic terminus, higher than that found for the methyl ester group of Fubinaca and for the *N*-methylamide group of LONI11.

The water permanency of LONI4 was more than 10% of the time, whereas for MDMB-Fubinaca and LONI11, it reached only 5%. The authors Michel et al. and Kumar et al. previously predicted the possible involvement of a water network formed by a water molecule strongly bound to the receptor, mediating the interactions among the ligand, His178 and Ser383 [47,68]. The capacity of the ligand to activate or deactivate the receptor could be largely due to its ability to form a specific water network. However, in the case of LONI4, the MD experiments demonstrated that the presence of a permanent negative charge allowed the carboxylic group to form strong polar interactions with three positive centers in the binding pocket represented by Arg182, Lys192 and Lys376 residues. This binding mode seemed to be preferred over the water network and the related interactions with Ser383 and His178. This difference is fundamental to explain the inverse agonist activity of LONI4. Moreover, the aromatic portion of the molecule seems to prefer a different accommodation in the receptor pocket. Thus, the novel compound LONI11 bearing the *N*-methyl-amide *C*-terminus and the 2,4-dichloro-benzyl ring largely interacted with the CB1 binding cavity, as in the case of MDMB-Fubinaca. On the contrary, LONI4 (bearing a free carboxylic acid) was not able to strongly interact with the polar key residues His178 and Ser383 either directly or through a water network; instead, it may have been able to establish a whole new set of polar interactions with the positive residues present at the receptor binding cavity (Lys376, Lys192 and Arg182), leading to the inactivation of the CB1 receptor.

## 5. Conclusions

In the last two decades, obesity has assumed the form of a pandemic, often related to the development of cardiovascular diseases (CVD) and strokes. There is a worldwide emergency that requires the development of novel and safer anti-obesity treatments. Rimonabant and sibutramine associated with lifestyle modifications were successfully engaged in the control of obesity by reducing appetite and weight gain. Their complex action mechanism promotes monoamine reuptake in the central nervous system (CNS), the secretion of neuropeptides with anorexigenic activity and the lowering of those with orexigenic properties, the elevation of metabolism, and the improvement of the peripheral sympathetic activity. Though those drugs have shown to be effective to control body weight and to manage obesity, they have been withdrawn from the market due to their important side effects. Thus, a novel drug able to provide the same therapeutic efficacy without the dangerous side effects is an urgent need. An agonist able to bind a specific receptor that preferentially drives a single downstream signaling cascade could represent a possible solution. However, the broad distribution of cannabinoid receptors in different areas of the body allows them to interact with a variety of system networks. The CB1 receptors are connected with different receptor-activity modulatory proteins, and the possibility to assume a selectively targetable ligand-binding conformation is still uncertain; currently, there are few process and site-specific CB1 targeted drugs. 

**Figure 9:**
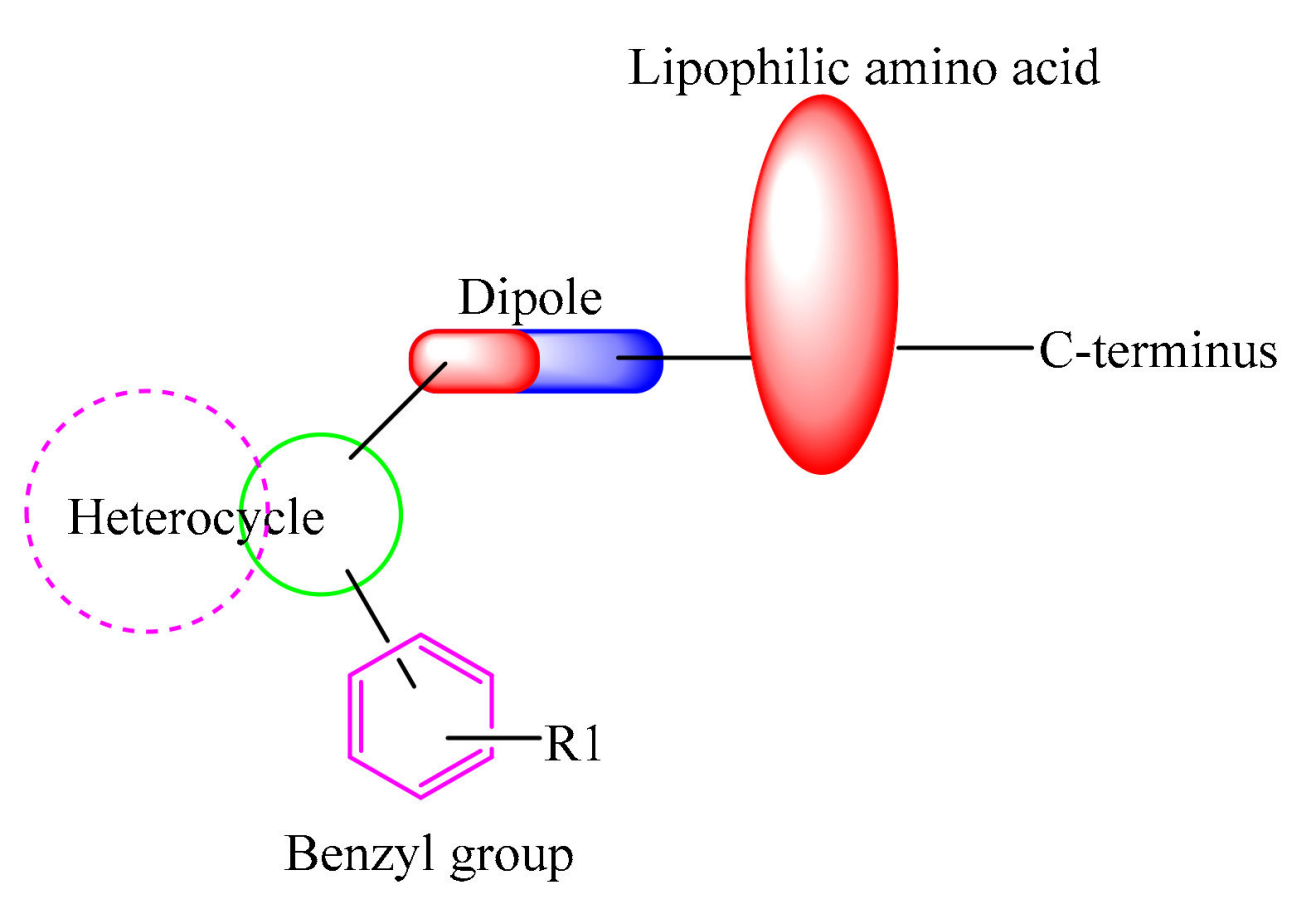
Structural features of the synthetic cannabinoids reported in this study.

Our study identified a series of lonidamine joined Leu, *tert*-Leu and Val amino acids with different *C*-terminal functional groups (LONI1-4,11) as novel compounds endowed with orexant/anorexant activity. They are structurally related to the Fubinaca series and follow the same general structure reported by Schoeder et al. [30]. However, in this study, novel features were identified. In particular, the four structural features described by Huffman et al. [69,70] were well defined in our compounds, namely: (i) An heterocyclic scaffold represented by an indole, indazole or imidazole ring with several substitutions; (ii) a dipolar linker such as an amide or ester; (iii) a lipophilic moiety such as the side chain of an amino acid or a lipophilic chain; and (iv) a hydrophobic side chain bound to the heterocyclic scaffold. This general structure was only able to establish the structural requirements for CB1 receptor affinity without taking intrinsic activity into consideration. In this work, we have demonstrated the importance of the *C*-terminus group, which is shown in the general structure depicted in Figure 9. Polar and uncharged *C*-terminus groups such as methyl ester and methyl amide moiety are able to act as H bond donors and are required for agonist activity, while a more polar *C*-terminal amide group is detrimental, giving weak agonists [33]. The free carboxylic group at *C*-terminus is able to shift agonist to antagonist activity. However, a large structure-activity relationships (SAR) study should be designed in order to verify our hypothesis.

Since one of them (LONI11) exhibited a potent agonist activity at CB1, the antinociceptive activity was also analyzed and revealed a good analgesic effect after sub-cutaneous and intracerebroventricular administration in vivo and a significative anti-inflammatory effect after s.c. administration, suggesting potential activity at the periphery. Even if the preliminary biological data in vivo and in vitro of these novel compounds were promising, a deep investigation on animal models is necessary to assess their selectivity, potency and long-term effects upon chronic and acute administration. These results could be useful as a starting point to optimize the pharmacological properties of rimonabant/Fubinaca hybrids and to better understand the molecular mechanism below their biological profile. An understanding of all the neuroendocrine networks and their roles in the hypothalamus to regulate the feeding behavior could lead to the development of novel and safer approaches to manage the main nutritional disorders. 

## 6. Patents

This work is part of the European patent EP18170728, deposited on 4th May 2018 by some of the authors involved in this work with the approval of all the inventors.

## Figures and Tables

**Table 1:** Binding affinity (K_i_) and signal properties efficacy (E_max_) and potency (EC_50_) of lonidamine-based compounds.

Compounds	Sequence	K_i_ (nM)	[^35^S]GTPγS Binding	
			E_max_ (%)	EC_50_ (nM)
WIN55,212-2		10 ± 1	173 ± 11	56 ± 3.8
JWH-018 [33]		3.5 ± 1	163 ± 5.2	16 ± 3
LONI1 [33]	Lonidamine-*tert*-Leu-OH	0.08 *	84 ± 6.6	>1 μM
LONI2 [33]	Lonidamine-*tert*-Leu-OCH_3_	3.1 *	143 ± 5.7	8.4
LONI3 [33]	Lonidamine-*tert*-Leu-NH_2_	17 *	139 ± 4.5	126
LONI4 [33]	Lonidamine-Val-OH	2.6 *	82 ± 10.6	>1 μM
LONI10	Lonidamine-Val-NHMe	84 ± 3.4	100 ± 1.7	n.r.
LONI11	Lonidamine-*tert*-Leu-NHMe	11 ± 1.2	151 ± 3	200 ± 13
LONI12	Lonidamine-Leu-NHMe	320 ± 16	101 ± 1	n.r.

K_i_ values were calculated from the corresponding displacement curves of Figure 2A. The E_max_ and EC_50_ were extrapolated from the dose–response curves of Figure 2B. Data represent the mean ±S.E.M. from least three independent experiments. n.r.: Not relevant. * Mean of three independent experiments (S.D. values are in the range of 5%–10%).

## Supplementary Materials

The following are available online at https://www.mdpi.com/2218-273X/9/9/492/s1, RP-HPLC analytical traces, ^1^H NMR and LRMS for the final compounds, molecular modeling and MD details.
