# Supplementary material for: Discovery of Orexant and Anorexant Agents with Indazole Scaffold Endowed with Peripheral Antiedema Activity
Source: Biomolecules. 2019 Sep 16;9(9):492. doi: 10.3390/biom9090492 (PMC6770484; doi:10.3390/biom9090492)

DISCOVERY OF OREXANT AND ANOREXANT AGENTS WITH INDAZOLE SCAFFOLD ENDOWED WITH PERIPHERAL ANTIEDEMA ACTIVITY

Marilisa, P. Dimmito[1], Azzurra Stefanucci[1,*], Stefano Pieretti[2], Paola Minosi[2], Szabolcs Dvorácskó[3], Csaba Tömböly[3], Gokhan Zengin[4], Adriano Mollica[1]

[1] Department of Pharmacy, University of Chieti-Pescara "G. d'Annunzio", Via dei Vestini 31, 66100 Chieti, Italy.

[2] Istituto Superiore di Sanità, Centro Nazionale Ricerca e Valutazione Preclinica e Clinica dei farmaci, Viale Regina Elena 299, 00161 Rome, Italy.

[3] Institute of Biochemistry, Biological Research Centre of the Hungarian Academy of Sciences, Temesvári krt. 62. 6726 Szeged, Hungary.

[4] Department of Biology, Science Faculty, Selcuk University, Konya, Turkey.

Corresponding author: a.stefanucci@unich.it

Table of contents	Pages
Analytical RP-HPLC traces **LONI 10-12**	S2
LRMS **LONI10-12**	S3-S5
^{1}H NMR **LONI10-12**	S6-S8
**LONI10-12** characterization	S9
Molecular modeling and MD details	S10-S13

Analytical RP-HPLC traces

LONI 10: Loni Val-NH-CH_3

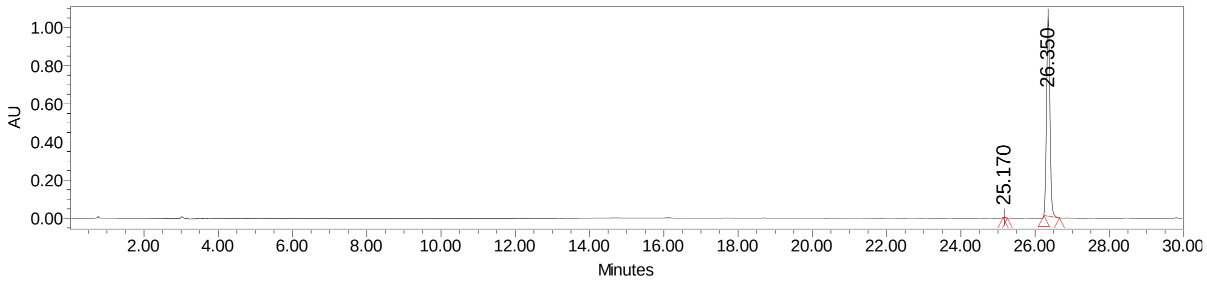

	Retention Time	Area	% Area	Height
1	25.170	12506	0.19	3115
2	26.350	6545480	99.81	1039046

LONI 11: Loni *tert*-Leu-NH-CH_3

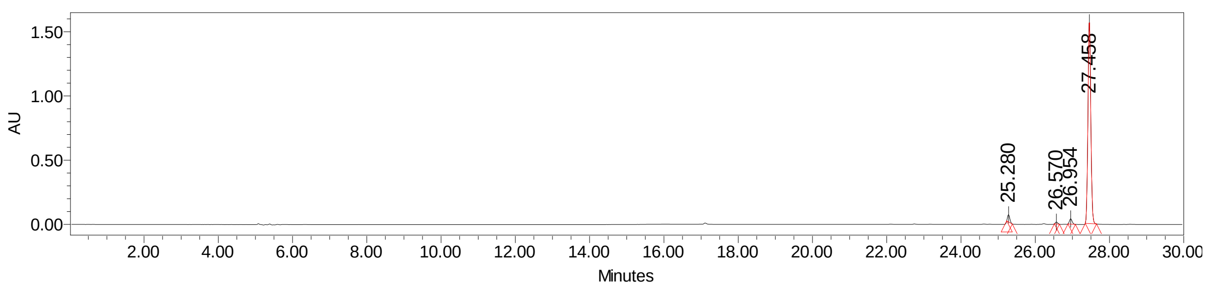

	Retention Time	Area	% Area	Height
4	27.458	8457289	95.17	1573124
3	26.954	203285	2.27	40579
2	26.570	62830	0.40	14578
1	25.280	229110	2.26	54476

LONI 12: Loni Leu-NH-CH_3

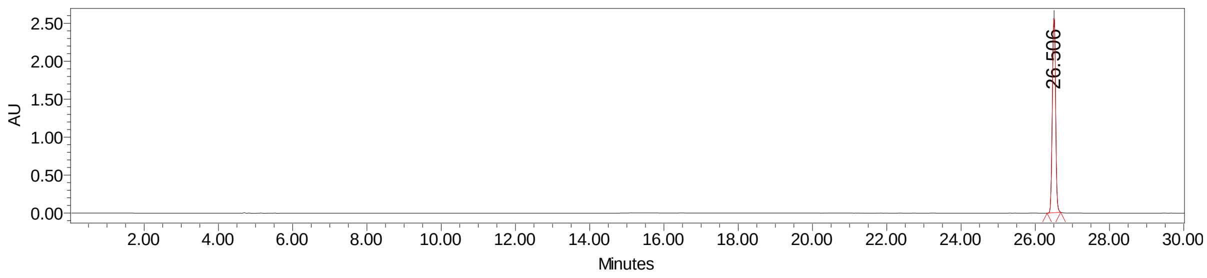

LRMS LONI 10

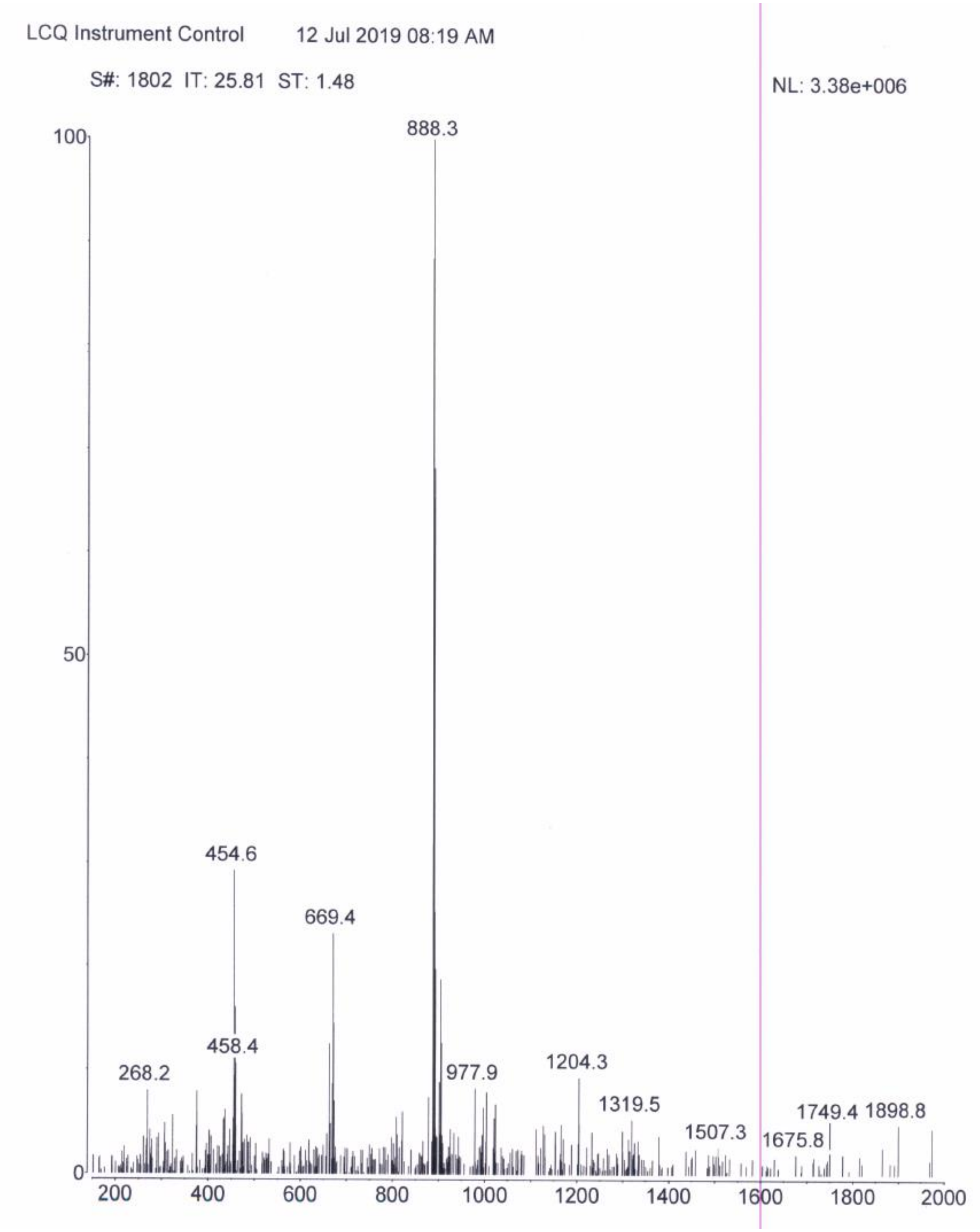

LCQ Instrument Control
12 Jul 2019 08:19 AM
S#: 1802 IT: 25.81 ST: 1.48
NL: 3.38e+006
100
50
0
888.3
454.6
669.4
458.4
268.2
977.9
1204.3
1319.5
1507.3
1675.8
1749.4
1898.8
200
400
600
800
1000
1200
1400
1600
1800
2000

LRMS LONI 11

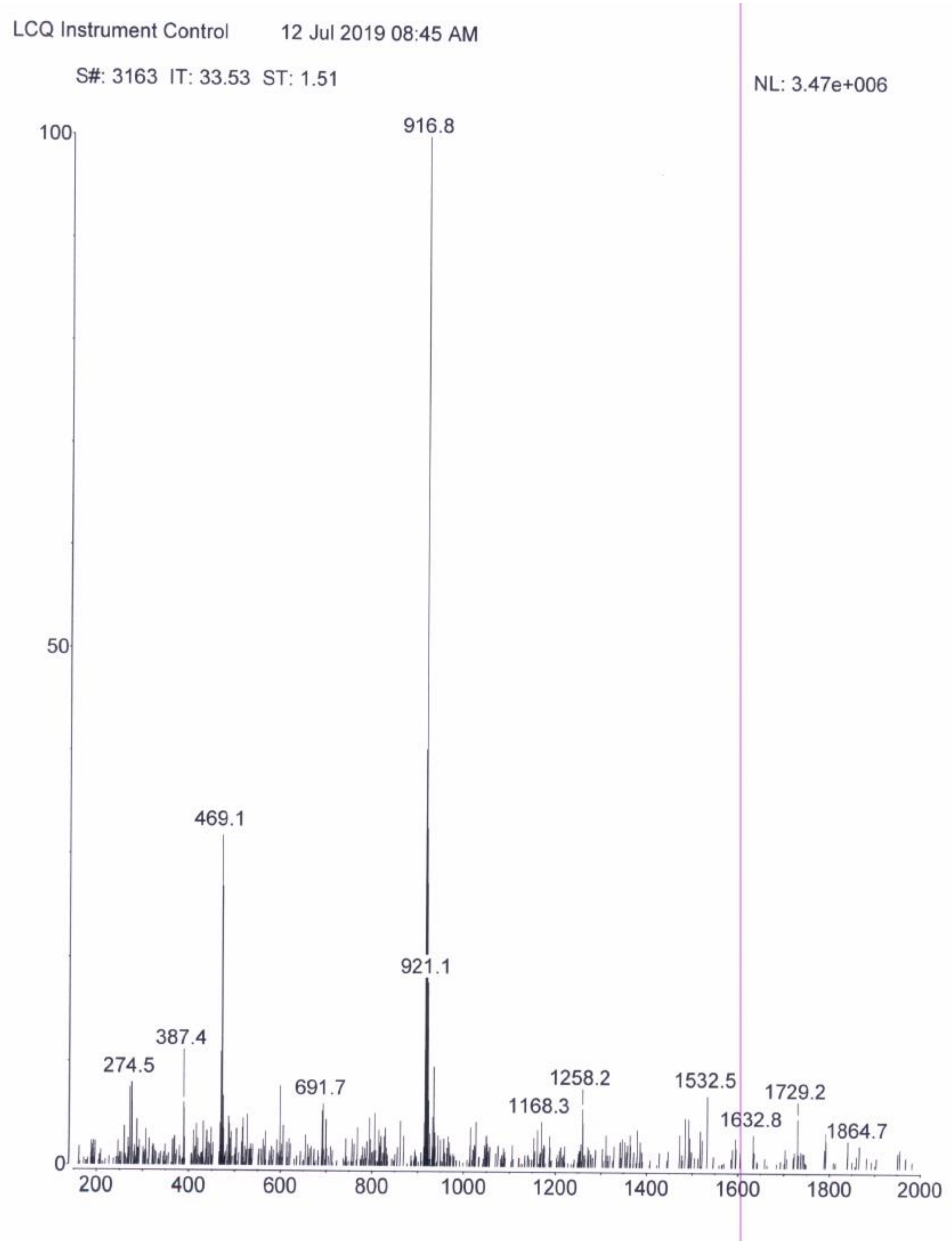

LCQ Instrument Control
12 Jul 2019 08:45 AM
S#: 3163 IT: 33.53 ST: 1.51
NL: 3.47e+006
100
50
0
916.8
469.1
921.1
387.4
274.5
691.7
1168.3
1258.2
1532.5
1632.8
1729.2
1864.7
200
400
600
800
1000
1200
1400
1600
1800
2000

LRMS LONI 12

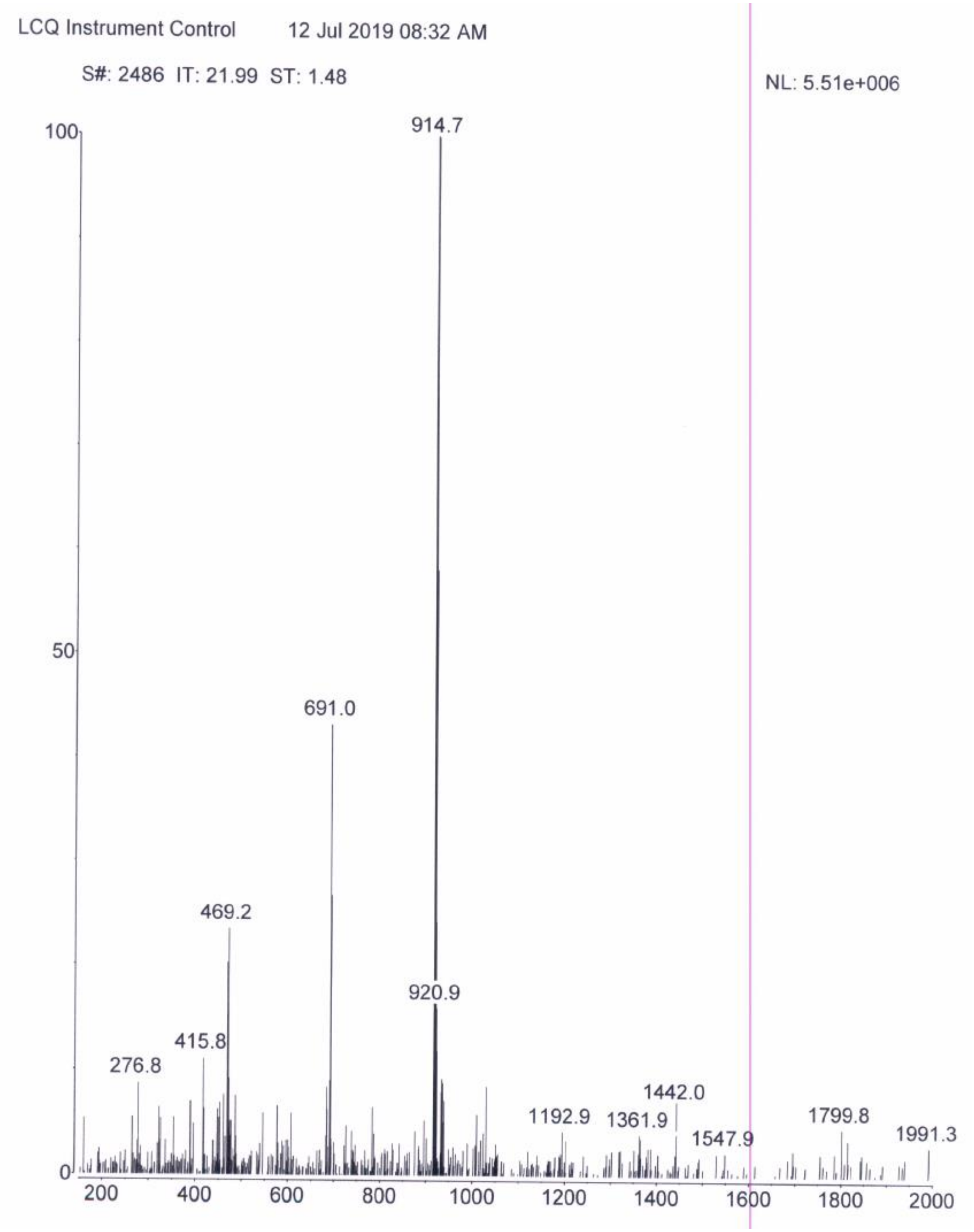
LCQ Instrument Control
12 Jul 2019 08:32 AM
S#: 2486 IT: 21.99 ST: 1.48
NL: 5.51e+006
100
50
0
914.7
691.0
469.2
920.9
415.8
276.8
1442.0
1192.9
1361.9
1547.9
1799.8
1991.3
200
400
600
800
1000
1200
1400
1600
1800
2000

^{1}H NMR LONI 10 in DMSO-d$_6$

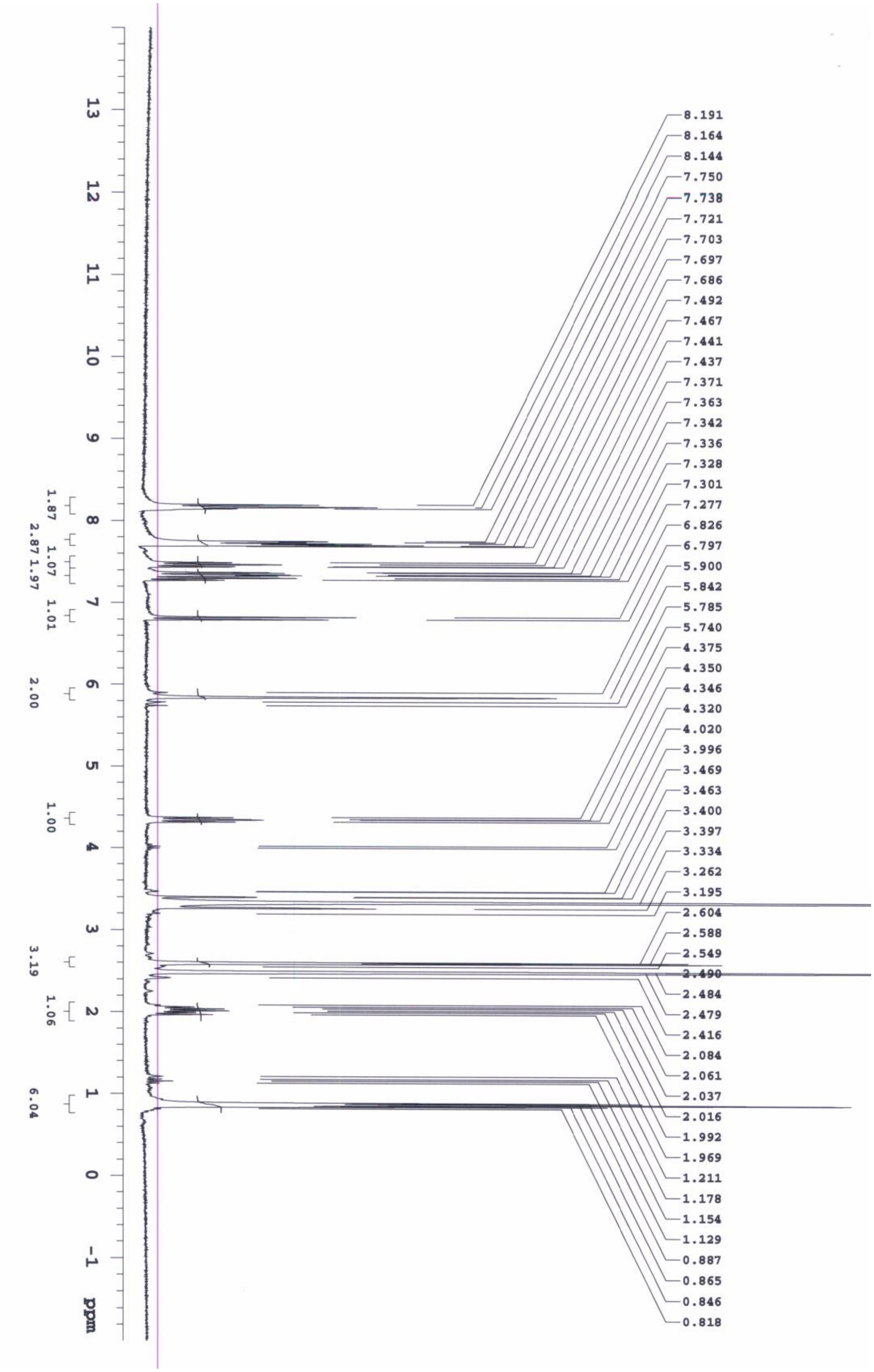

^{1}H NMR LONI 11 in DMSO-d_6

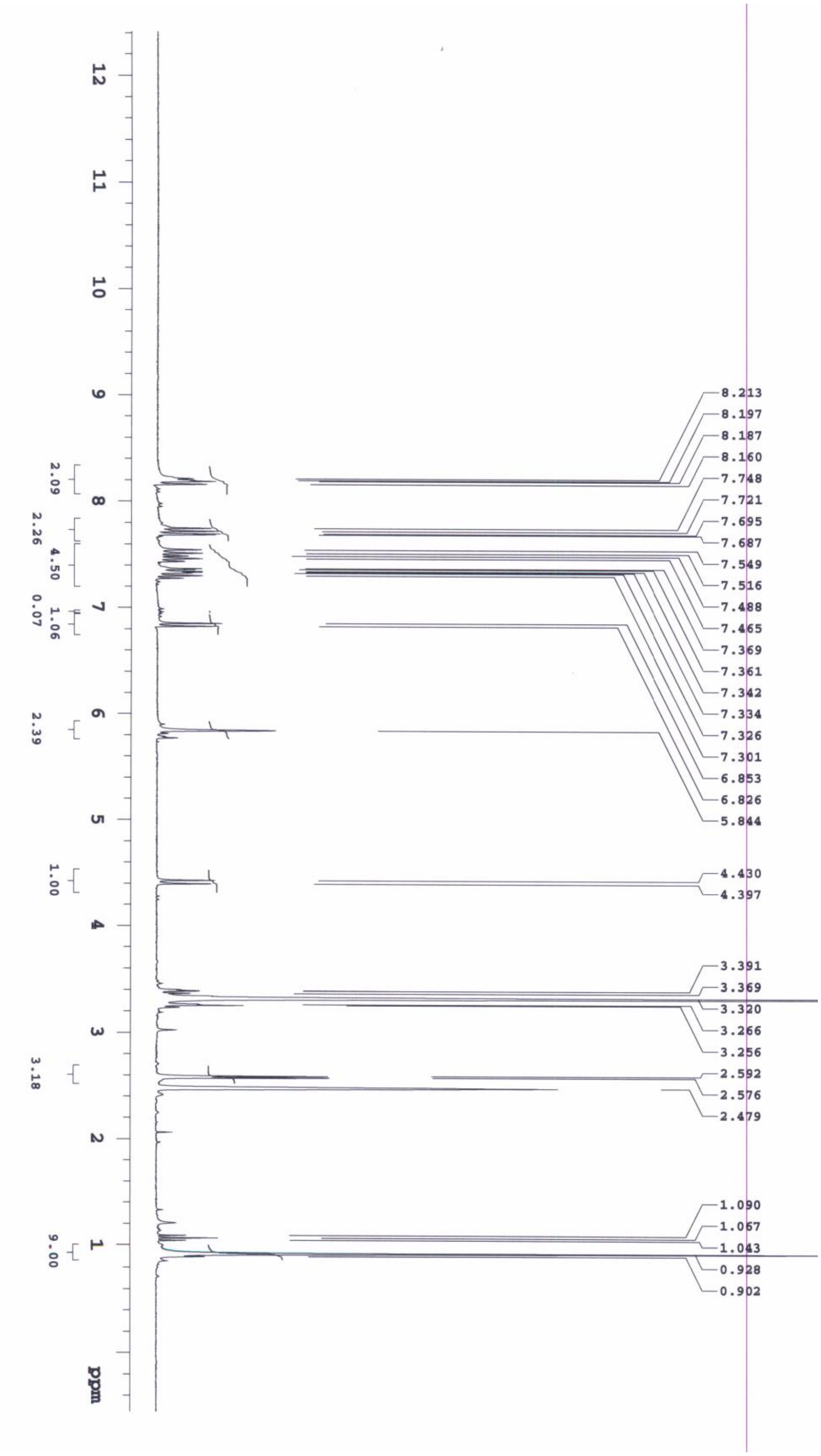

^{1}H NMR LONI 12 in DMSO-d_6

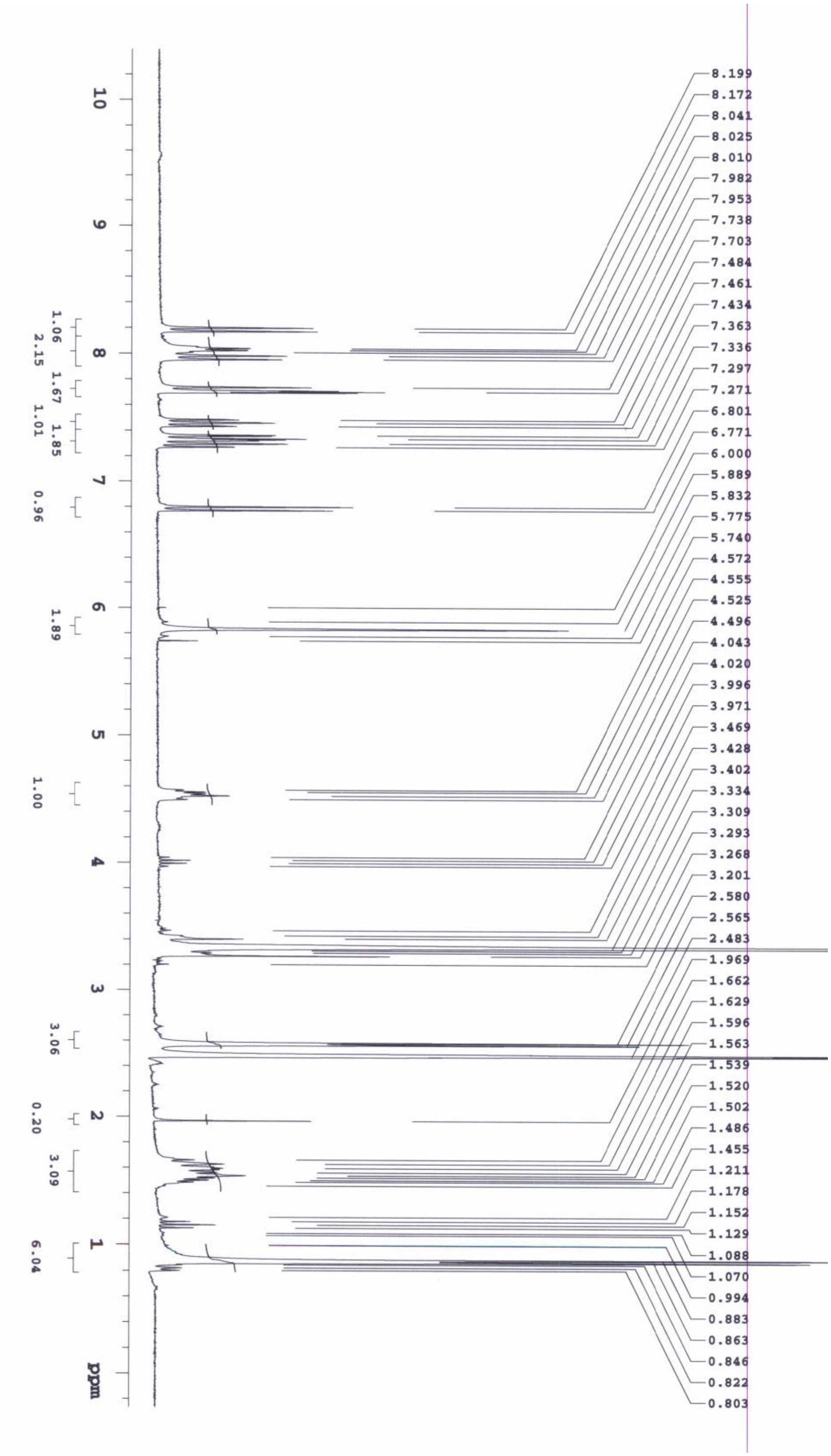

LONI10-12 characterization:

(S)-1-(2,4-dichlorobenzyl)-*N*-(3-methyl-1-(methylamino)-1-oxobutan-2-yl)-1H-indazole-3-carboxamide (LONI10). Compound LONI4 was transformed in the *N*-methyl amide derivative LONI10 following the general procedure. The desired compound was obtained in 96% yield, after reaction work up. Rt (analytical HPLC) = 26.35 min. ^{1}H NMR (300 MHz, DMSO-d_6) δ 8.19-8.14 (m, 2H, H1 and NH amide), 7.75-7.68 (m, 3H, H4, N<u>H</u>(CH_3), H-3), 7.49-7.27 (m, 3H, H-2, H2, H3), 6.81 (d, 1H, H-1), 5.84 (s, 2H, -CH_2-), 6.77 (d, 1H, H7), 5.84 (s, 2H, H-1), 4.35 (t, 1H, CH$^\alpha$ Val), 2.56 (d, 3H, NH-C<u>H</u>$_3$ Val), 2.05 (m, 1H, <u>CH</u>(CH_3)$_2$ Val), 0.85 (dd, 6H, CH_3x2 Val). ^{13}C NMR (300 MHz, DMSO-d_6) δ 171.5, 161.5, 141.5, 137.9, 133.7, 133.4, 130.8, 129.5, 128.2, 127.7, 123.3, 122.5, 122.2, 110.9, 57.7, 50.1, 49.1, 31.5, 25.8, 19.6, 18.7. LRMS calcd. for $C_{21}H_{22}Cl_2N_4O_2$: 432.1; found: 454.6 $[M+Na]^+$.

(S)-1-(2,4-dichlorobenzyl)-*N*-(3,3-dimethyl-1-(methylamino)-1-oxobutan-2-yl)-1H-indazole-3-carboxamide (LONI11). Compound LONI1 was transformed in the *N*-methyl amide derivative LONI11 following the general procedure. The desired compound was obtained in 97% yield, after reaction work up. Rt (analytical HPLC) = 27.45 min. ^{1}H NMR (300 MHz, DMSO-d_6) δ 8.21-8.16 (m, 2H, H1 and NH amide), 7.74-7.68 (m, 2H, H4 and N<u>H</u>(CH_3) *tert*-Leu), 7.54-7.46 (m, 2H, H2 and H-3), 7.36-7.30 (m, 2H, H3 and H-2), 6.84 (d, 1H, H-1), 5.84 (s, 2H, -CH_2-), 4.42 (d, 1H, CH$^\alpha$ *tert*-Leu), 2.58 (d, 3H, NH(C<u>H</u>$_3$) *tert*-Leu), 0.92 (s, 9H, CH_3x3 *tert*-Leu). ^{13}C NMR (300 MHz, DMSO-d_6) δ 170.5, 160.7, 141.5, 137.7, 133.7, 133.5, 130.8, 129.5, 128.2, 127.7, 123.4, 122.4, 122.2, 110.1, 59.3, 49.1, 35.2, 26.9, 25.6. LRMS calcd. for $C_{22}H_{24}Cl_2N_4O_2$: 446.1; found: 469.1 $[M+Na]^+$.

(S)-1-(2,4-dichlorobenzyl)-*N*-(4-methyl-1-(methylamino)-1-oxopentan-2-yl)-1H-indazole-3-carboxamide (LONI12). Compound LONI7 was transformed in the *N*-methyl amide derivative LONI12 following the general procedure. The desired compound was obtained in quantitative yield, after reaction work up. Rt (analytical HPLC) = 26.50 min. ^{1}H NMR (300 MHz, DMSO-d_6) δ 8.18 (d, 1H, H1), 8.04-7.95 (m, 2H, H4 and N<u>H</u>(CH_3)), 7.73-7.70 (m, 2H, H-3 and NH amide), 7.46 (t, 1H, H2), 7.36-7.27 (m, 2H, H-2 and H3), 6.79 (d, 1H, H-1), 5.83 (s, 2H, -CH_2-), 4.55 (q, 1H, CH$^\alpha$ Leu), 2.57 (d, 3H, NH(C<u>H</u>$_3$) Leu), 1.66-1.45 (m, 3H, CH_2^β Leu and CH$^\gamma$ Leu), 0.87 (dd, 6H, CH_3x2 Leu). ^{13}C NMR (300 MHz, DMSO-d_6) δ 172.6, 161.7, 141.4, 138.1, 133.8, 133.2, 130.7, 129.5, 128.2, 127.7, 123.2, 122.6, 122.3, 110.8, 51.2, 50.1, 49.1, 41.7, 26.1, 24.8, 23.3, 22.1. LRMS calcd. for $C_{22}H_{24}Cl_2N_4O_2$: 446.1; found: 469.2 $[M+Na]^+$.

Molecular modeling and MD details

Figure 1S. (A) MDMB-Fubinaca crystallographic pose (light blue) superimposed to the same self-docked ligand (light brown) by Glide. (B) Superimposition of MDMB-Fubinaca (light blue), LONI11 (violet), LONI4 (light brown) poses.

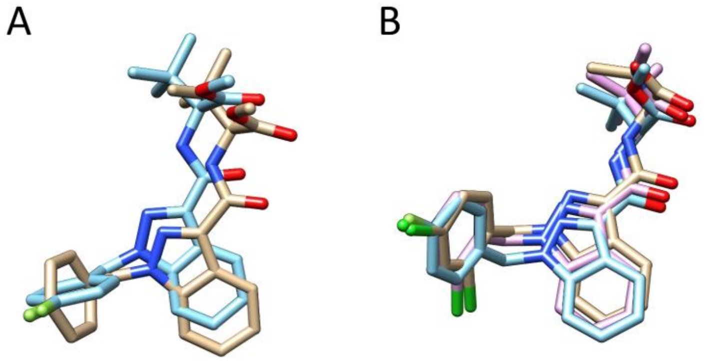

Figure 2S. 2D interaction diagram of (A) MDMB-Fubinaca, (B) LONI11 and (C) LONI4 docked to CB1 receptor (pdb id: 6N4B).

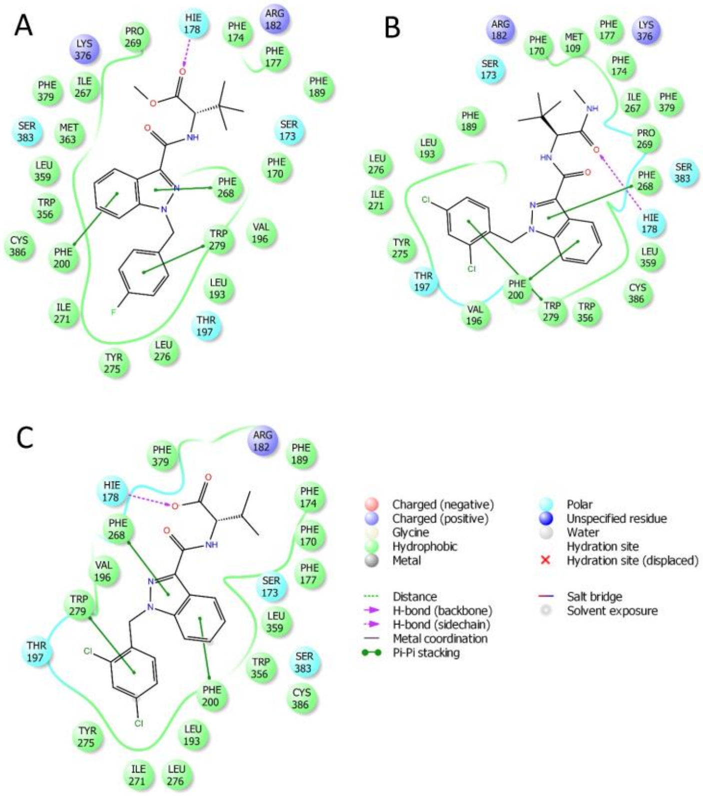

Figure 3S. System model used in the molecular dynamic simulation.

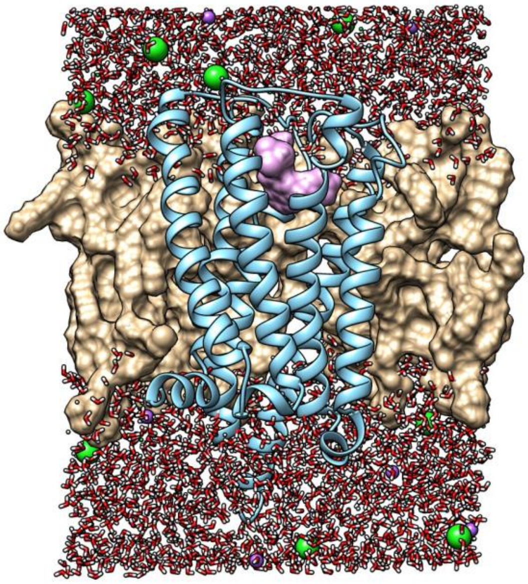

Figure 4S. Protein-ligand interactions of CB1 receptor with (A) MDMB-Fubinaca ligand, (B) LONI4 and (C) LONI11, calculated after 20 ns of MD.

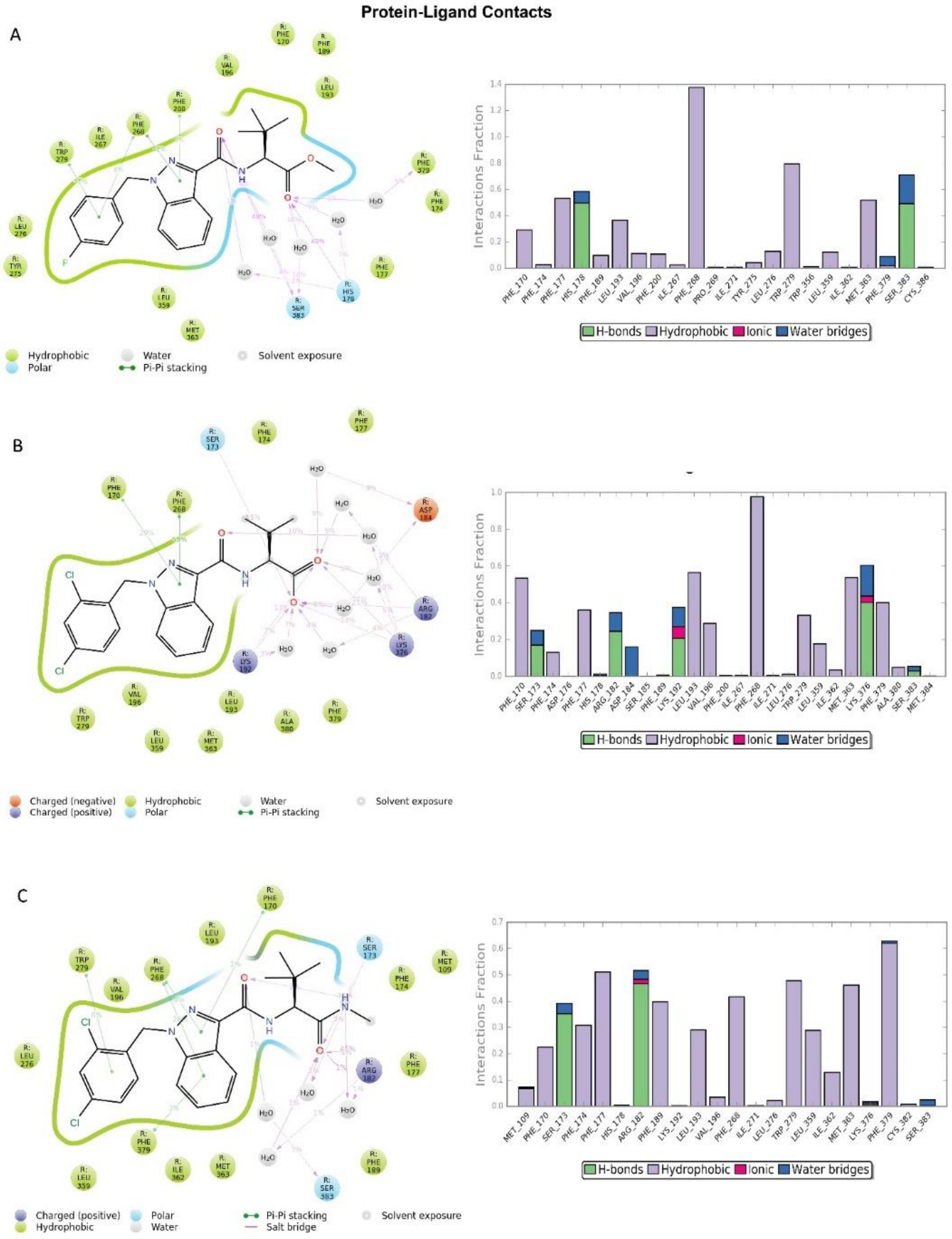

Figure 5S. RMSD (angstroms) for ligand MDMB-Fubinaca (A), LONI4 (B) and LONI11 (C) and the receptor CB1 during 20 ns of molecular dynamic simulation.

A

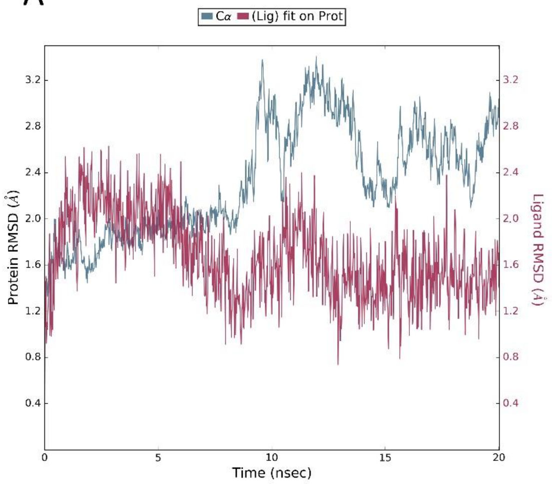

B

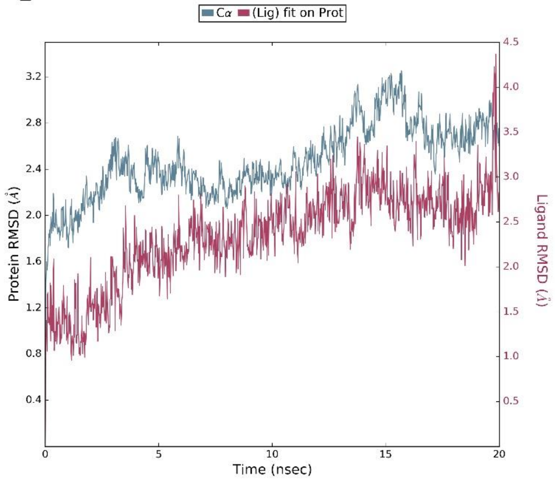

C

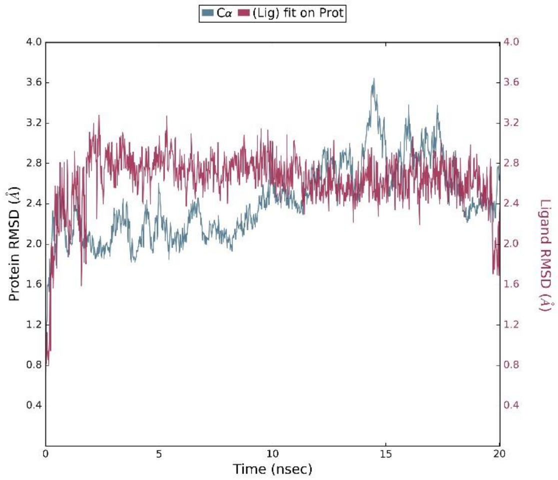

Supplement: Supplementary file 1 [file biomolecules-09-00492-s001.pdf]
